# *Drosophila* carrying epilepsy-associated variants in the vitamin B6 metabolism gene *PNPO* display allele- and diet-dependent phenotypes

**DOI:** 10.1073/pnas.2115524119

**Published:** 2022-02-25

**Authors:** Wanhao Chi, Atulya S. R. Iyengar, Wenqin Fu, Wei Liu, Abigayle E. Berg, Chun-Fang Wu, Xiaoxi Zhuang

**Affiliations:** ^a^Committee on Genetics, Genomics and Systems Biology, University of Chicago, Chicago, IL 60637;; ^b^Department of Neurobiology, University of Chicago, Chicago, IL 60637;; ^c^Department of Biology, College of Liberal Arts and Sciences, University of Iowa, Iowa City, IA 52242;; ^d^Iowa Neuroscience Institute, University of Iowa, Iowa City, IA 52242;; ^e^Department of Environmental Health, School of Public Health, China Medical University, Shenyang 110122, China;; ^f^Grossman Institute for Neuroscience, Quantitative Biology and Human Behavior, University of Chicago, Chicago, IL 60637

**Keywords:** gene–environment interaction, phenotypic variation, pyridox(am)ine 5*^′^*-phosphate oxidase, vitamin B6, sugarlethal

## Abstract

Pyridox(am)ine 5*^′^*-phosphate oxidase (PNPO) catalyzes the rate-limiting step in the synthesis of pyridoxal 5*^′^*-phosphate (PLP), the active form of vitamin B6 required for the synthesis of neurotransmitters gamma-aminobutyric acid (GABA) and the monoamines. Pathogenic variants in *PNPO* have been increasingly identified in patients with neonatal epileptic encephalopathy and early-onset epilepsy. These patients often exhibit different types of seizures and variable comorbidities. Recently, the *PNPO* gene has also been implicated in epilepsy in adults. It is unclear how these phenotypic variations are linked to specific *PNPO* alleles and to what degree diet can modify their expression. Using CRISPR-Cas9, we generated four knock-in *Drosophila* alleles, *h^WT^*, *h^R^*^116^*^Q^*, *h^D^*^33^*^V^* , and *h^R^*^95^*^H^*, in which the endogenous *Drosophila PNPO* was replaced by wild-type human *PNPO* complementary DNA (cDNA) and three epilepsy-associated variants. We found that these knock-in flies exhibited a wide range of phenotypes, including developmental impairments, abnormal locomotor activities, spontaneous seizures, and shortened life span. These phenotypes are allele dependent, varying with the known biochemical severity of these mutations and our characterized molecular defects. We also showed that diet treatments further diversified the phenotypes among alleles, and PLP supplementation at larval and adult stages prevented developmental impairments and seizures in adult flies, respectively. Furthermore, we found that h^R95H ^had a significant dominant-negative effect, rendering heterozygous flies susceptible to seizures and premature death. Together, these results provide biological bases for the various phenotypes resulting from multifunction of *PNPO*, specific molecular and/or genetic properties of each *PNPO* variant, and differential allele–diet interactions.

## Significance

Both genetic and environmental factors contribute to epilepsy. Understanding their contributions and interactions helps disease management. However, it is often challenging to study gene–environment interaction in humans due to their heterogeneous genetic background and less controllable environmental factors. The fruit fly, *Drosophila melanogaster*, has been proven to be a powerful model to study human diseases, including epilepsy. We generated knock-in flies carrying different epilepsy-associated pyridox(am)ine 5*^′^*-phosphate oxidase (*PNPO*) alleles and studied the developmental, behavioral, electrophysiological, and fitness effects of each mutant allele under different dietary conditions. We showed that phenotypes in knock-in flies are allele and diet dependent, providing clues for timely and specific diet interventions. Our results offer biological insights into mechanisms underlying phenotypic variations and specific therapeutic strategies.

Pyridox(am)ine 5*^′^*-phosphate oxidase (PNPO; Enzyme Commission Number 1.4.3.5) is a rate-limiting enzyme in the synthesis of vitamin B6 (VB6) (1). Mutations in PNPO can cause neonatal epileptic encephalopathy, a devastating disease that usually leads to death if untreated (2). Recently, PNPO mutations have also been reported in patients with infantile spasms and early-onset epilepsy (3–6), and the *PNPO* gene has been recognized as 1 of the 16 epilepsy genes involved in genetic generalized epilepsy in adults (7). The increasingly recognized impact of *PNPO* in epilepsy raises the question of how *PNPO* variants are implicated in different types of epilepsy and ages of seizure onset.

VB6 comprises pyridoxine, pyridoxamine, pyridoxal, and their corresponding phosphorylated forms (i.e., pyridoxine 5*^′^*-phosphate, pyridoxamine 5*^′^*-phosphate, and pyridoxal 5*^′^*-phosphate [PLP]). Among them, PLP is the only active form, which is a cofactor for more than 140 enzymes, including those required for the synthesis of the neurotransmitters dopamine, serotonin, and gamma-aminobutyric acid (GABA) (8). Unlike plants, bacteria, and fungi, humans and other animals cannot synthesize VB6 de novo, instead relying on dietary VB6, which is converted to PLP by PNPO (1). The amino acid sequence and structure of PNPO are conserved well from *Escherichia coli* to humans (9–11). In mammals, *PNPO* is highly expressed in the liver, kidney, and brain (12). The functional PNPO protein is a homodimer, and it binds to two molecules of flavin mononucleotide (FMN) as cofactors (13–15).

To date, more than 30 *PNPO* variants have been identified in neonatal epileptic encephalopathy and other epilepsy patients since the first report in 2005 (2, 16, 17). Biochemical studies show that different variants reduce the PNPO enzymatic activity to different levels, ranging from 0 to 83% (2, 3, 18, 19). Such a wide range of variation can be attributed to the differential effects of different mutations on the catalytic site, FMN binding, and/or protein folding and thermostability (17, 20, 21). While these in vitro studies show clear evidence that different PNPO mutations affect enzymatic activity to varying degrees, they do not explain the variation in seizure types, seizure onsets, and comorbidities manifested by PNPO-deficient patients carrying the same mutation (3, 17). Furthermore, *PNPO* variants have never been studied in vivo for their molecular or functional defects. It remains unknown whether they can also indirectly affect the gene’s function at transcriptional and/or translational levels. These molecular characterizations are crucial for understanding the phenotypic variation from a molecular perspective. Lastly, the dietary contribution to the phenotypic variations has not been systematically examined. Therefore, there is a need to develop in vivo systems to better understand individual *PNPO* variants, their potential contributions to phenotypic variations, and the effect of diet on the phenotype expression associated with each variant.

*Drosophila melanogaster* has been proven to be a valuable model system to study human diseases, including epilepsy (22–26). Our previous studies have also demonstrated that the *PNPO* gene is functionally conserved between humans and flies; similar to humans, PNPO deficiency in *Drosophila* also leads to seizures and premature death (27). Here, we generated four *Drosophila* knock-in (KI) strains carrying either wild-type (WT) human pyridox(am)ine 5*^′^*-phosphate oxidase (*hPNPO*) allele (*h^WT^*) or one of three *hPNPO* epilepsy-associated alleles *h^R^*^116^*^Q^*, *h^D^*^33^*^V^* , and *h^R^*^95^*^H^*. We examined KI flies at the molecular, circuitry, behavioral, and organismal levels. In addition to the reported impaired enzymatic activity, we found that each mutant variant conferred a specific molecular effect; *h^D^*^33^*^V^* decreased messenger RNA (mRNA) level, *h^R^*^95^*^H^* reduced protein stability, and *h^R^*^116^*^Q^* altered the protein localization of *PNPO* in the brain. Furthermore, we observed a wide range of phenotypes in KI flies, including developmental impairments, behavioral hyper- or hypoactivity, spontaneous seizure discharges or abnormal firing patterns, and shortened life span. The phenotypic variation is associated with the known biochemical severity of these mutations and our characterized molecular defects. We also showed that diet treatments further diversified the phenotypes among alleles, and PLP supplementation at larval or adult stages prevented developmental impairments and seizures in adult flies, respectively. Finally, we found that *h^R^*^95^*^H^* had a significant dominant-negative effect, and heterozygous flies were prone to seizures upon electroconvulsive stimulation and showed increased lethality on the VB6-devoid diet.

## Results

### Generation of KI *Drosophila* Strains Carrying h*PNPO* Variants Identified from Epilepsy Patients.

**Fig. 1.**
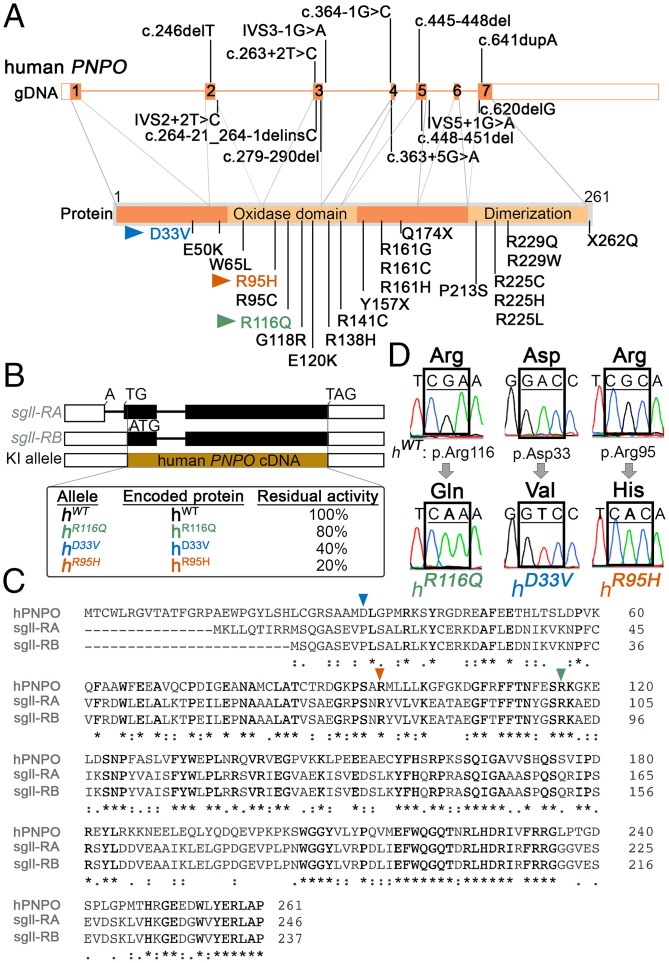
Generation of *Drosophila* KI alleles carrying *hPNPO* epilepsy-associated variants. (*A*) The *hPNPO* gene and epilepsy-associated variants. Exons are annotated based on the most common form of transcript (Ensembl transcript ENST00000642017.2). Different variants identified in epilepsy patients are shown. Three variants examined in this study are in blue, green, and orange. gDNA, genomic DNA. (*B*) The *Drosophila PNPO* gene, *sgll*, encodes two transcript forms *sgll-RA* and *sgll-RB*. Both of them are expressed; however, *sgll-RB* is globally expressed and has a relatively high expression level in comparison with *sgll-RA* (29). The protein products of these two forms share the C terminus but differ in the N terminus, with the sgll-RA form containing nine extra amino acids (30). Given that the first intron in *sgll-RA* may play a regulatory role in gene expression level and/or pattern, *sgll-RB* is replaced by *hPNPO* cDNAs in the KI alleles. Black boxes indicate exons, white boxes indicate untranslated regions (UTRs), and black lines indicate introns. KI alleles corresponding to WT, R116Q, D33V, and R95H are designated as *h^WT^*, *h^R^*^116^*^Q^*, *h^D^*^33^*^V^* , and *h^R^*^95^*^H^*, respectively. The residual activities of corresponding *hPNPO* mutant proteins were previously measured biochemically in vitro (3, 18). (*C*) Amino acid sequence alignment analysis. Semiconserved (.), conserved (:), and absolutely conserved (*) are indicated. Three amino acids mutated in this study are marked with triangles. (*D*) The presence of the targeted mutation in each KI allele is confirmed by sequencing chromatograms.

*hPNPO* spans ∼8 kbp in the genome and has 17 splice variants (28). The most ubiquitously expressed variant has seven exons and encodes a 261-amino acid protein (Fig. 1*A*). In PNPO, two domains are required for the enzymatic activity; one is the oxidase domain located in the middle region, and the other one is the dimerization domain located at the C terminus. Functional PNPO is a dimer; the monomer has no enzymatic activities (14).

The *Drosophila PNPO* gene (*sugarlethal* [*sgll*]) encodes two protein products using alternative transcriptional starts; they share the C terminus and differ at the N terminus, with one form containing nine extra amino acids (Fig. 1 *B* and *C*) (10). The protein sequence of *Drosophila* PNPOs shares ∼45% identity and 75% similarity with hPNPO (Fig. 1*C*). Functionally, PNPO deficiency in flies causes seizures and premature death as it does in humans (16, 27). Both seizures and premature death can be rescued by ubiquitous expression of WT *hPNPO* (16, 27), suggesting that the molecules and signaling pathways underlying PNPO deficiency-induced seizures and premature death are conserved between humans and flies. Thus, *Drosophila* can be used as an in vivo system to study *hPNPO* variants identified in patients.

To date, more than 30 different *hPNPO* variants have been identified in neonatal epileptic encephalopathy patients and patients with early-onset epilepsy (Fig. 1*A*). Although these mutations tend to spread across the *hPNPO* gene/protein, more variants are associated with oxidase and dimerization domains.

Here, we generated four KI *Drosophila* alleles using CRISPR-Cas9, in which the *Drosophila PNPO* gene, *sgll* (10), was replaced by *hPNPO* cDNAs (Fig. 1*B*). These four KI alleles were designated as *h^R^*^116^*^Q^*, *h^D^*^33^*^V^* , *h^R^*^95^*^H^*, and *h^WT^*. The three mutant alleles, *h^R^*^116^*^Q^*, *h^D^*^33^*^V^* , and *h^R^*^95^*^H^*, were chosen to represent different severities of PNPO deficiency based on in vitro biochemical studies of their corresponding mutant proteins (h^R116Q ^, h^D33V ^, and h^R95H ^hereafter). The residual enzymatic activities of h^R116Q ^, h^D33V ^, and h^R95H ^are ∼80, 40, and 20% of h^WT ^, respectively (3, 18). The mutation in each allele was confirmed by Sanger sequencing (Fig. 1*D*). All KI alleles were initially balanced over a *TM6B, Hu, Tb* chromosome (*TM6B* hereafter) to circumvent potential homozygous lethality.

### Distinct Molecular Alterations Linked to Different *hPNPO* Epilepsy- Associated Variants.

**Fig. 2.**
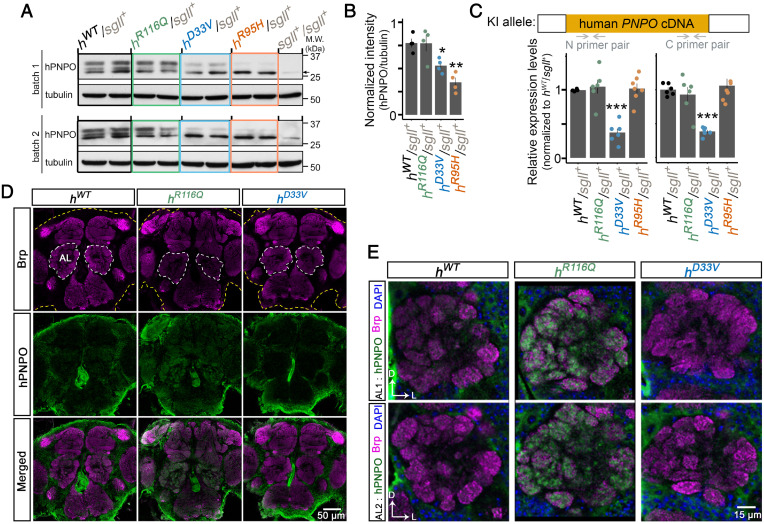
Molecular characterization of each KI allele. (*A*) Western blot of hPNPO in an adult head homogenate from each KI/*sgll*^+^ line. *n* = 4 per genotype. In each batch, one sample from *w^1118^* flies (*sgll^+^*/*sgll^+^*) is loaded as the antibody control. Tubulin is the loading control. The arrow indicates a band also seen in *w*^1118^ flies, which most likely is a nonspecific band (*SI Appendix*, Fig. S3). M.W.: molecular weight. (*B*) Quantification of hPNPO in *A* (the top band). Error bars represent mean ± SEM. The two-tailed Student’s *t* test with Bonferroni’s correction compared with *h^WT^*/*sgll*^+^ was used. *P < 0.05; **P < 0.01. (*C*) Quantification of the mRNA level of *hPNPO* in adult heads by qRT-PCR. Two primer pairs are used to target the N and C terminus of *hPNPO* cDNA. *n* = 4 to 7 per primer pair per genotype. Error bars represent mean ± SEM. The two-tailed Student’s *t* test with Bonferroni’s correction compared with *h^WT^*/*sgll*^+^ was used. ***P < 0.001. (*D*) Immunohistochemistry staining of hPNPO in the adult brain of *h^WT^*, *h^R^*^116^*^Q^*, and *h^D^*^33^*^V^* homozygotes. The *h^R^*^95^*^H^* line is not included due to the lethality of *h^R^*^95^*^H^* homozygotes in the normal diet and the greatly reduced hPNPO protein level in their heads (*A*). (*E*) hPNPO staining in the ALs of *h^WT^*, *h^R^*^116^*^Q^*, and *h^D^*^33^*^V^* homozygotes. D, dorsal; L, lateral.

Other than reducing the enzymatic activity (2, 3, 18, 19), little is known about the molecular characteristics of *hPNPO* variants. Thus, after establishing the KI lines, we first studied whether these epilepsy-associated variants could affect *hPNPO* expression and localization using homozygous flies (*SI Appendix*, Figs. S1 and S2). The *h^R^*^95^*^H^* allele was not included initially because *h^R^*^95^*^H^* homozygotes were lethal (see below). To include all four KI alleles, we further crossed KI lines with *w^1118^* to generate KI/*sgll^+^* flies and performed western blotting and qRT-PCR using adult heads. We confirmed that the antibody for hPNPO did not recognize *Drosophila* PNPO (*SI Appendix*, Fig. S3). Results from western blot demonstrated that the R116Q mutation did not change the hPNPO protein level, whereas D33V and R95H significantly reduced it (Fig. 2 *A* and *B* and *SI Appendix*, Fig. S1 *A* and *B*). Interestingly, multiple bands instead of a single band appeared on the blot. The molecular nature of these bands is unclear. One possibility is that given only that the *sgll-RB* form was replaced in the KI allele (Fig. 1*B*), a chimeric hPNPO protein could also be expressed, which contains the nine extra amino acids from sgll-RA in the front of the hPNPO protein. It is also possible that the multiple bands were due to protein modifications or proteolysis, as suggested in a previous study (14).

The reduced hPNPO protein level associated with D33V or R95H mutations could be due to changes at the transcriptional or translational level. We, therefore, examined the mRNA level of *hPNPO* in each genotype using qRT-PCR. Two pairs of primers that specifically targeted the N- or C-terminal regions of *hPNPO* cDNA were used. Results from both primer pairs showed that D33V but not R95H led to a reduced mRNA level (Fig. 2*C* and *SI Appendix*, Fig. S1*C*). The reduced mRNA level associated with D33V was observed in flies reared on both normal and sugar-only diets (*SI Appendix*, Fig. S2), suggesting that it is likely intrinsic to D33V. The mechanism of D33V in mediating the change is unclear.

Next, we examined the localization of hPNPO protein in the adult brain using immunohistochemistry staining with an anti-hPNPO antibody. Anti-Bruchpilot (Brp) antibody was used to visualize synapse-rich neuropils (31). We found that h^WT ^was ubiquitously expressed in the brain with the strongest staining in the cell body rind, which comprises neuronal cell bodies and certain types of glial cells (Fig. 2*D* and *SI Appendix*, Fig. S4*A*) (32). The antibody is presumably specific to hPNPO as very faint staining was observed in *w^1118^* control flies (*SI Appendix*, Fig. S5). There was little overlap between Brp and hPNPO staining, suggesting that h^WT ^is not enriched in the terminal structures. Consistently, no hPNPO staining was observed in fiber bundles, such as anterior optic tract, inferior fiber system, and medial antennal lobe (AL) tract (*SI Appendix*, Fig. S4*A*). Moderate hPNPO staining was also shown in areas surrounding neuropils. One prominent region is the area surrounding the AL (Fig. 2*D*). The ALs are the primary centers for olfactory processing in flies, and they are organized into individual glomeruli. Each glomerulus contains synapses formed by three types of neurons: olfactory receptor neurons (ORNs), local interneurons (LNs), and projection neurons (PNs). While the cell bodies of ORNs are far away from ALs, the cell bodies of LNs and PNs surround ALs. The PNs extend their dendrites to glomeruli and receive excitatory input from ORNs to relay olfactory information to higher brain centers, such as the mushroom body calyx and the lateral horn of the protocerebrum; the LNs are interneurons, and most of them are GABAergic (33). Subcellularly, hPNPO mainly stayed in the cell bodies of surrounding neurons (*SI Appendix*, Fig. S4*B*). Furthermore, the staining of hPNPO did not overlap with DAPI, a nucleus marker, suggesting that hPNPO is mainly cytosolic. Notably, compared with the strong staining of hPNPO in cell bodies, faint hPNPO staining was also observed in the glomeruli region. However, it does not seem to overlap with the Brp staining (*SI Appendix*, Fig. S4*B*). Thus, the hPNPO staining in the glomerulus region was most likely from the dendrites of LNs or glial cells.

A similar hPNPO staining pattern was observed in h^D33V ^brains (Fig. 2*D*), suggesting that D33V does not affect the protein localization in the brain. In striking contrast, strong terminal staining for hPNPO was detected in h^R116Q ^brains (Fig. 2 *D* and *E*). The increased staining appeared to occur in all terminal structures in the brain, with the most dramatic change in the AL (*SI Appendix*, Fig. S6). Since the hPNPO protein level in *h^R^*^116^*^Q^* is comparable with that in *h^WT^* (Fig. 2 *A* and *B*), we speculate that the increased terminal staining is most likely caused by altered localization of h^R116Q ^in the brain, at least in some cell types.

Taken together, molecular characterization of *hPNPO* variants in KI flies demonstrates that in addition to reducing the enzymatic activity as shown in previous studies (2, 3, 18, 19), *hPNPO* variants can also affect the gene’s function through altering the mRNA and/or protein level or the protein localization in the brain.

### Distinct Developmental Effects Associated with Different *hPNPO* Variants.

All balanced KI lines were viable and fertile. However, no homozygous KI adult flies were observed from *h^R^*^95^*^H^*/*TM6B* breeding bottles. To rule out the contribution of any off-target mutations, the KI line was backcrossed to *w^1118^* for five generations. No homozygous *h^R^*^95^*^H^* pupae or adult flies were observed after backcross, suggesting that the lethality was most likely due to the R95H mutation in hPNPO.

**Fig. 3.**
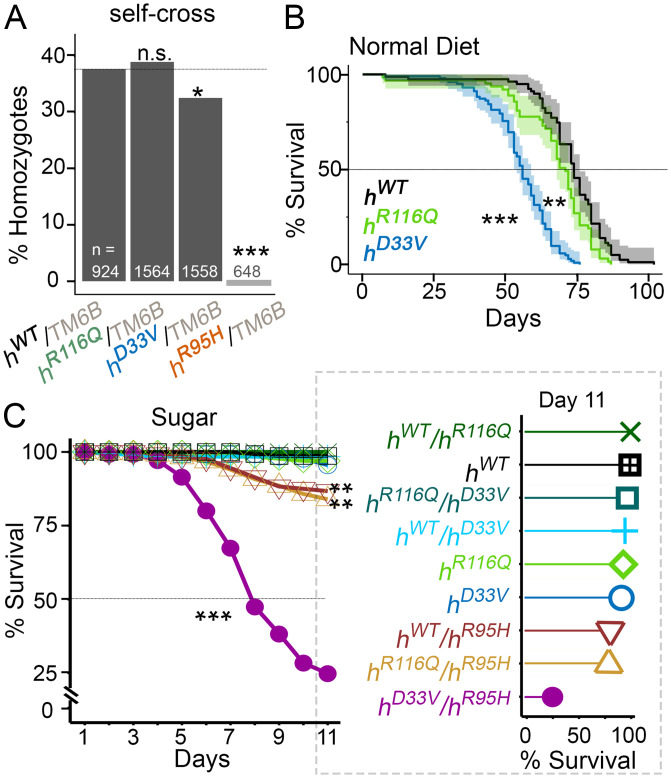
Developmental and survival phenotypes of KI flies from various genotypes. (*A*) Generation of homozygous flies from each KI line. The *χ*^2^ test of homogeneity compared with *h^WT^/TM6B* (the gray line) is shown. (*B*) Survival of homozygous flies on the normal diet. *n* = 63 to 102, log-rank test compared with *h^WT^*. (*C*) Survival of flies from various genotypes on the sugar-only diet. *n* = 102 to 218, log-rank test compared with *h^WT^*. Survival of each genotype on day 11 is also shown in the lollipop plot in *Right*. n.s., *P* > 0.05. *P < 0.05; **P < 0.01; ***P < 0.001.

To systematically study the effects of the R95H mutation as well as the two other mutations on development, we self-crossed each KI line and examined the number of homozygous KI flies in the F1 generation. The ratio of KI homozygous flies in all flies (homozygosity ratio) was further calculated. Consistent with our initial observations, no *h^R^*^95^*^H^* homozygotes were observed (Fig. 3*A*). A significantly decreased homozygosity ratio was also found from *h^D^*^33^*^V^* self-breeding. By contrast, the homozygosity ratio in *h^R^*^116^*^Q^* was comparable with that in *h^WT^*. Thus, these three mutant alleles have differential effects on development. This conclusion was corroborated by complementation tests (*SI Appendix*, Fig. S7).

### Allele-Dependent Diet Modifications of Life Span in KI Flies.

PLP is involved in a variety of biological processes (8), yet the potential cumulative effect of chronic PNPO deficiency has never been studied. We examined whether *h^R^*^116^*^Q^* and *h^D^*^33^*^V^* could affect the survival of adult flies on the normal diet. Survival data showed that *h^R^*^116^*^Q^* had a slightly shortened life span compared with *h^WT^* (median: 71 vs. 74 d for *h^R^*^116^*^Q^* and *h^WT^*, respectively; *P* < 0.01) (Fig. 3*B*). In comparison, the life span of *h^D^*^33^*^V^* was much shorter (median: 56 d; *P* < 0.001). The lethality of *h^R^*^116^*^Q^* and *h^D^*^33^*^V^* flies correlated well with the residual enzymatic activity of *h^R^*^116^*^Q^* and *h^D^*^33^*^V^* measured by in vitro studies (3, 18). Together, these studies demonstrate that even mild PNPO deficiency can have a long-term deleterious consequence, even in the presence of dietary VB6.

We have previously reported that PNPO-deficient flies (*sgll^95^*) are short lived on the sugar-only diet (i.e., VB6-devoid diet) (27), suggesting that the sugar-only diet is useful for exacerbating PNPO deficiency. We thus generated homozygous (two same KI alleles), heterozygous (one *h^WT^* allele and one mutant allele), and transheterozygous (two mutant alleles) flies using four KI alleles and examined their survival on sugar. We found that the most dramatic lethal phenotype among these nine genotypes was from *h^D^*^33^*^V^* /*h^R^*^95^*^H^* flies (Fig. 3*C*), of which ∼75% died by day 11. The significant lethality in *h^D^*^33^*^V^* /*h^R^*^95^*^H^* flies was not surprising since based on in vitro residual enzymatic activity studies, *h^D^*^33^*^V^* /*h^R^*^95^*^H^* has the most severe PNPO deficiency. We also observed lethality from *h^R^*^116^*^Q^*/*h^R^*^95^*^H^* and *h^WT^*/*h^R^*^95^*^H^* flies; about 15% of them died by day 11 (Fig. 3*C*), suggesting that *h^R^*^95^*^H^* is indeed the most severe allele among all three mutant alleles. The fact that *h^WT^*/*h^R^*^95^*^H^* showed lethality suggests that *h^R^*^95^*^H^* may cause haploinsufficiency or have a dominant-negative effect, which is unexpected because *PNPO* has been considered as autosomal recessive in heritance (https://omim.org). These two possibilities were further studied (see below).

Overall, these studies demonstrate that both *PNPO* variants and dietary conditions can affect survival of KI flies.

### Allele-Dependent Diet Modifications of Locomotor Behaviors of KI Flies.

**Fig. 4.**
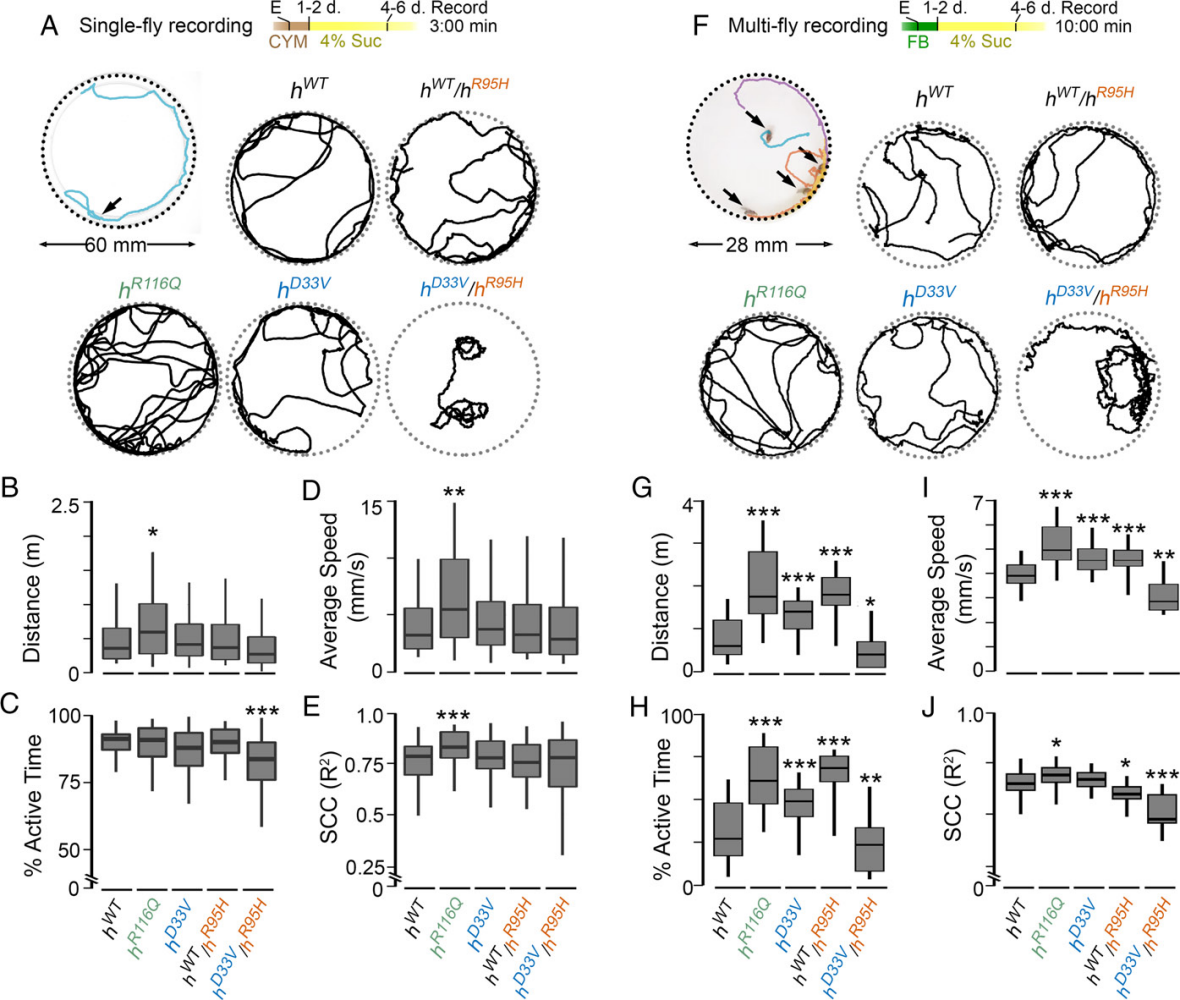
Behavioral analyses of KI flies in an open-field arena. (*A* and *F*) Breeding and testing conditions and representative tracks from each genotype. Flies were eclosed (E) on either the CYM or FB diet and then, transferred to the sugar-only diet (4% Suc). (*B–E*) Total distance traveled, percentage of active time, average speed, and SCC (*Materials and Methods* has calculation details), respectively, of flies from various genotypes generated and tested in *A*. *n* = 50 to 75 flies. (*G–J*) Total distance traveled, percentage of active time, average speed, and SCC, respectively, of flies from various genotypes generated and tested in *F*. *n* = 28 to 44 flies. The two-tailed Student’s *t* test with Bonferroni’s correction compared with *h^WT^* was used. *P < 0.05; **P < 0.01; ***P < 0.001.

The *h^R^*^116^*^Q^* and *h^D^*^33^*^V^* homozygous flies did not exhibit noticeable behavioral deficits when reared on the normal diet (the cornmeal–yeast–molasses [CYM] media). Consistent with this observation, mutant homozygous flies traveled a similar total distance in an open-field arena, with comparable speed, percentage of active time, and speed correlation coefficient (SCC) compared with *h^WT^* homozygotes (*SI Appendix*, Fig. S8*A*–*E*). However, when eclosed flies were reared on sugar for 4 to 6 d (Fig. 4*A*), *h^R^*^116^*^Q^* homozygotes exhibited hyperactivity; they traveled farther than *h^WT^* and had an increased average speed (Fig. 4 *B–E*). The increased average speed and total traveled distance were not observed in *h^D^*^33^*^V^* homozygotes in the same breeding and testing conditions (Fig. 4 *B–E*). Interestingly, when files were bred on the other standard diet, the Frankel & Brosseau’s (FB) media, in which yeast extract and nonfat dry milk were used to replace the dried yeast in the CYM media (34, 35), both *h^R^*^116^*^Q^* and *h^D^*^33^*^V^* homozygous flies exhibited hyperactivity (*SI Appendix*, Fig. S8*F*–*J*). The difference was maintained when eclosed flies were further reared on sugar for 4 to 6 d (Fig. 4 *F–J*). Therefore, diet plays a significant role in modifying the behaviors of *h^R^*^116^*^Q^* and *h^D^*^33^*^V^* homozygous flies.

Since we observed lethality in *h^WT^*/*h^R^*^95^*^H^* and *h^D^*^33^*^V^* /*h^R^*^95^*^H^* flies when they were reared on sugar (Fig. 3*C*), we further analyzed their behavior under different breeding and testing dietary conditions. When bred on the CYM media, *h^WT^*/*h^R^*^95^*^H^* flies behaved similarly to *h^WT^* homozygotes in both sugar-only and CYM testing conditions (Fig. 4 *B–E* and *SI Appendix*, Fig. S8*B*–*E*). The *h^D^*^33^*^V^* /*h^R^*^95^*^H^* flies, however, were less active in both testing conditions, and some even exhibited tortious walking paths when reared on sugar (a representative track is in Fig. 4*A*) and consequently, had low SCCs. The average SCC of *h^D^*^33^*^V^* /*h^R^*^95^*^H^* flies is, however, comparable with that of *h^WT^* (Fig. 4*E*). The fact that the *h^D^*^33^*^V^* /*h^R^*^95^*^H^* flies exhibited behavioral deficits even on the normal diet suggests that their residual PNPO enzymatic activity is insufficient to convert dietary VB6 to PLP to maintain normal behaviors.

When bred on the FB media, *h^WT^*/*h^R^*^95^*^H^* flies were more active than *h^WT^* homozygotes in both sugar-only and FB testing conditions (Fig. 4 *G–J* and *SI Appendix*, Fig. S8*G*–*J*), which resembles *h^R^*^116^*^Q^* and *h^D^*^33^*^V^* homozygotes in the same conditions. The different behaviors of *h^WT^*/*h^R^*^95^*^H^*, *h^R^*^116^*^Q^*, or *h^D^*^33^*^V^* flies on different media are likely due to the mild PNPO deficiency in them and its interaction with different diets. Consistent with this notion, *h^D^*^33^*^V^* /*h^R^*^95^*^H^* flies, which have much more severe PNPO deficiency than them, exhibited hypoactivity and tortious walking paths on both media (Fig. 4 and Movie S1).

Taken together, behavioral analyses of KI flies on different dietary conditions demonstrate that diet can potentially interact with PNPO deficiency to modify the locomotor behaviors of KI flies, and the effect of diet–allele interactions becomes prominent when flies have mild PNPO deficiency.

### Spontaneous Seizures in KI Flies with Severe PNPO Deficiency.

**Fig. 5.**
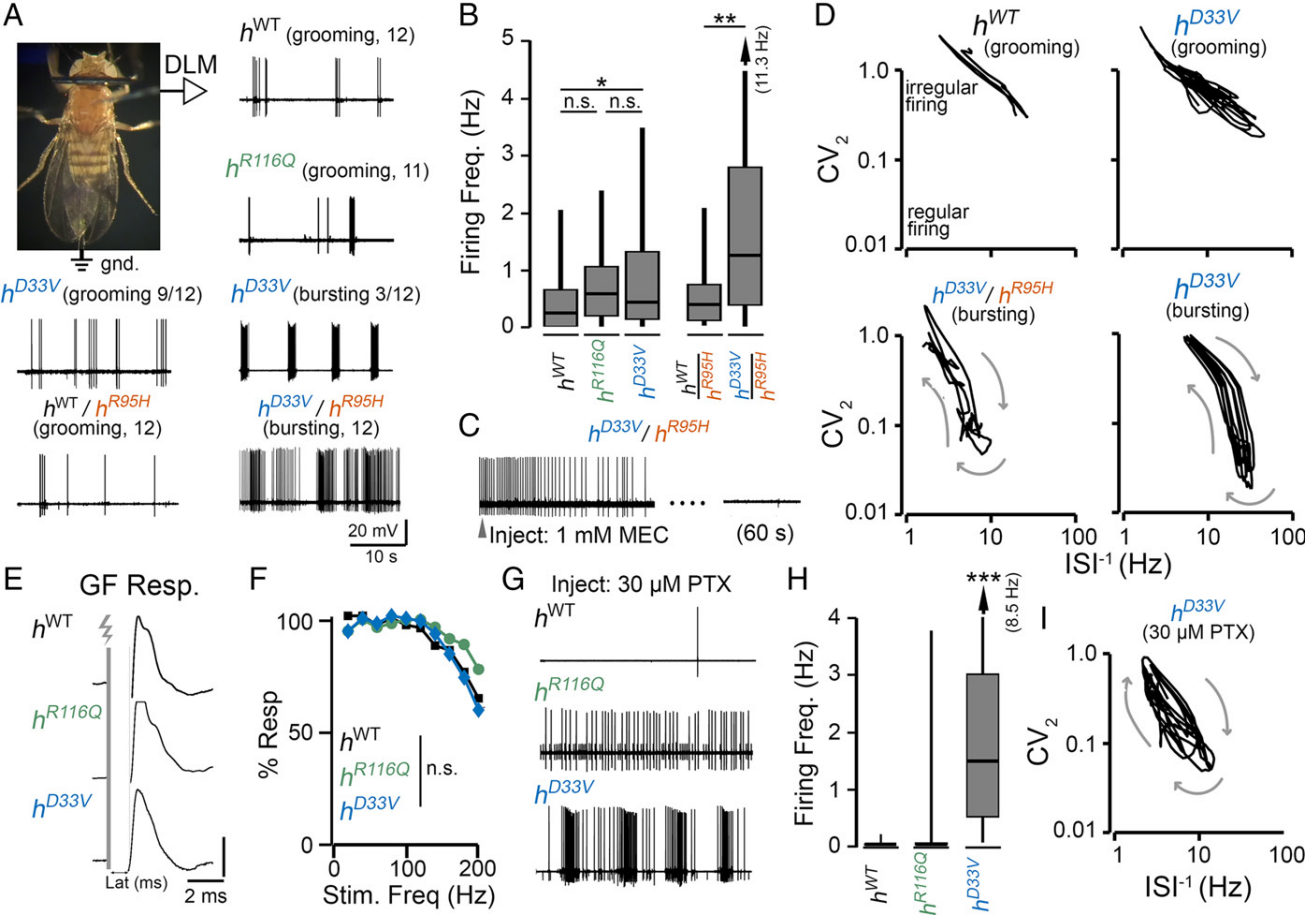
Electrophysiological correlates of seizures-related activity in KI flies. (*A*) Tethered fly preparation (*Materials and Methods* has details) and representative traces of spontaneous activity in DLM flight muscle spiking flies from each genotype reared on the sugar-only diet. Firing in *h^WT^*, *h^R^*^116^*^Q^*, and *h^D^*^33^*^V^* (the trace below the fly image) and in *h^WT^*/*h^R^*^95^*^H^* is related to grooming behavior. Abnormal rhythmic spike bursts are occasionally observed in *h^D^*^33^*^V^* (3 of 12 individuals), while aberrant spike discharges are observed in *h^D^*^33^*^V^* /*h^R^*^95^*^H^* flies. (*B*) Average DLM firing rate in flies from each genotype. The arrow indicates extension of the 95th percentile to the indicated values. *n* = 26 to 47 traces. (*C*) Acetylcholine is the primary excitatory neurotransmitter in the central nervous system (CNS) of *Drosophila*. Systemic injection of nicotinic acetylcholine receptor antagonist mecamylamine (MEC) blocks the spontaneous firing in *h^D^*^33^*^V^* /*h^R^*^95^*^H^*, indicating that the aberrant firing is generated from the CNS. (*D*) Firing pattern analyses. Plots of the instantaneous firing frequency (ISI^–1^) vs. the CV_2_ (a measure of firing regularity) readily distinguish grooming-related firing (*h^WT^* is shown) from self-similar bursting in *h^D^*^33^*^V^* and *h^D^*^33^*^V^* /*h^R^*^95^*^H^*. Plots from representative firing patterns are shown (*Materials and Methods* has details on construction of ISI^–1^–CV_2_ plots). (*E*) Representative DLM spikes triggered by stimulation of the GF escape circuit (gray bar and lightning bolt). Note the similar stimulus–response latency in the respective genotypes (∼1.4 ms). (*F*) GF circuit following ability. DLM spike response rate (% Resp) to trains of 10 GF stimuli at varying frequencies. For clarity, error bars are omitted. No significant differences between genotypes were observed. (*G*) DLM spiking evoked by injection of a low dose of the GABA_A _receptor blocker PTX (30 µM). Occasional spikes in *h^WT^* corresponded with grooming, the *h^R^*^116^*^Q^* individual selected showed repetitive DLM firing, and the *h^D^*^33^*^V^* flies displayed spike bursts. (*H*) Average DLM firing frequency following PTX injection (*n* = 18 to 39 traces from five to six flies per genotype). (*I*) ISI^–1^–CV_2_ plots of DLM firing in a representative *h^D^*^33^*^V^* fly following PTX injection. Kruskal–Wallis ANOVA rank-sum post hoc was used. n.s.: *P* > 0.05; *P < 0.05; **P < 0.01; ***P < 0.001.

The spectrum of behavioral phenotypes associated with KI lines prompted us to examine motor unit activity patterns and identify spontaneous seizure-associated spike discharges in the respective lines. We utilized a tethered fly preparation (Fig. 5*A*) to monitor dorsal longitudinal muscle (DLM) activity in intact, behaving flies. During flight, these muscles power the “down stroke” of the wing, and the isometric nature of their contractions facilitates prolonged recordings with minimal muscle damage. Importantly, the innervating DLM motor neuron (DLMn) serves as an output for several motor programs (e.g., flight, courtship song, and grooming activity) (26, 36–38). Several studies have utilized aberrant spiking in this motor unit to monitor spontaneous (39) and evoked (40) seizure activity, including our previous analyses of the original *sgll^95^* mutant (27). We found that *h^WT^* homozygotes reared on sugar displayed occasional grooming-related spiking (Fig. 5*A* and Movie S2), largely consistent with activity patterns in other control flies (41). In the *h^R^*^116^*^Q^* and *h^D^*^33^*^V^* mutants reared on the same diet, we mostly observed similar grooming-associated activity patterns, with a marginal increase in the average firing rate in *h^D^*^33^*^V^* flies compared with *h^WT^* controls (Fig. 5*B*). The increased average firing in *h^D^*^33^*^V^* flies was due to a small subset of *h^D^*^33^*^V^* individuals (3 of 12) showing a peculiar and highly stereotypic pattern of repeated DLM spike bursts (Fig. 5*A* and Movie S2). This bursting did not have clear behavioral correlates and was distinct from previously reported spontaneous seizure activity in *sgll^95^* mutants (27).

In contrast to the relatively mild phenotypes displayed by homozygous KI lines, we found ongoing spontaneous seizure activity in *h^D^*^33^*^V^* /*h^R^*^95^*^H^* transheterozygotes, manifesting as high-frequency spike burst discharges (Fig. 5*A* and Movie S2), with overall DLM firing rates for *h^D^*^33^*^V^* /*h^R^*^95^*^H^* substantially greater than *h^WT^* or *h^WT^*/*h^R^*^95^*^H^* flies reared on the same media (Fig. 5*B*). To determine if this aberrant spiking activity originated from the CNS or endogenously from the motor unit, we blocked central excitatory neurotransmission and monitored the effect on spiking activity. In flies, acetylcholine is the primary excitatory neurotransmitter in the CNS (42), while glutamate is the transmitter at the neuromuscular junction (43). We applied the nicotinic acetylcholine receptor blocker mecamylamine using a rapid systemic injection protocol (41) and found that the spike discharges in *h^D^*^33^*^V^* /*h^R^*^95^*^H^* were abolished (Fig. 5*C*), indicating that aberrant CNS activity drove the seizure-associated motor unit discharges in these flies.

During seizure-associated discharges in *h^D^*^33^*^V^* /*h^R^*^95^*^H^* mutants, bursts in *h^D^*^33^*^V^* , and grooming-associated activity in *h^WT^* and other KI flies, the DLM spiking intervals were highly variable, with instantaneous firing rates (defined as the reciprocal of the interspike interval [ISI^–1^]) ranging from ∼1 Hz to nearly 50 Hz. However, these activity patterns could be readily distinguished and were remarkably characteristic within mutant flies. To quantitatively delineate seizure-associated activity in KI flies from grooming-related spiking, we employed a nonlinear dynamical systems approach we had previously used to describe firing patterns in the *sgll^95^* mutants and other hyperexcitable flies (Fig. 5*D*) (27, 41). For each spike in the recording, we plotted the spike’s instantaneous firing rate (ISI^–1^) against the instantaneous coefficient of variation (CV_2_) (*Materials and Methods* has the definition). High CV_2_ values indicate irregular firing, while low values indicate rhythmic activity. Previously, we have shown that seizure-related bursting in *sgll^95^* mutants corresponded to a self-similar “looping” trajectory in the phase–space analysis, while grooming activity displayed a distinctive trajectory limited to high CV_2_ values (>0.5). Based on the ISI^–1^–CV_2_ plots (representative trajectories are shown in Fig. 5*D*), the orbiting trajectories of *h^D^*^33^*^V^* bursting and *h^D^*^33^*^V^* /*h^R^*^95^*^H^* spike discharges were readily distinguished from grooming-related behavior and from one another. Furthermore, the trajectories of *h^D^*^33^*^V^* /*h^R^*^95^*^H^* firing were qualitatively similar to spontaneous seizures in *sgll^95^* mutants (27), suggesting a shared neural mechanism underlying the aberrant activity.

### Reduced GABAergic Function in KI Mutant Flies.

Next, we asked what neurotransmission system(s) was responsible for the spontaneous seizures in *hPNPO* KI mutants. To provide an initial glance at excitatory cholinergic signaling, we examined properties of the giant fiber (GF) jump-and-flight escape pathway, a descending circuit with identified neurons as well as cholinergic, glutamatergic, and electrical synapses (44). Mutations disrupting neuronal excitability (45), cholinergic transmission (46), and gap junctions (47) lead to profound disruptions in GF pathway performance. In *h^WT^* flies, direct stimulation of the GF neuron leads to a DLM spike with a stereotypic latency of 1.41 ± 0.04 ms (Fig. 5*E*), similar to previously reported WT and control strains (i.e., “short latency” responses) (45, 48). We found that the GF latencies in *h^R^*^116^*^Q^* and *h^D^*^33^*^V^* were comparable with *h^WT^* (1.42 ± 0.08 and 1.46 ± 0.04 ms, respectively) (Fig. 5*E*). Furthermore, using a repetitive stimulation protocol across a wide range of frequencies (20 to 200 Hz), we found that *h^R^*^116^*^Q^* and *h^D^*^33^*^V^* were able to follow high-frequency stimulation to a similar degree as *h^WT^* (Fig. 5*F*). Taken together, these observations suggest that the function of both excitatory cholinergic transmission and electrical synapses is largely maintained in the KI mutants.

We further asked whether the PNPO mutations led to changes in GABAergic signaling given that PLP, the product of PNPO, is required for the GABA synthesis. In WT flies, injection of the noncompetitive GABA_A _receptor antagonist picrotoxin (PTX; concentration >50 µM) induces a stereotypic sequence of flight-like rhythmic DLM spiking (∼10 Hz) followed by spike bursts exceeding 100 Hz (41, 49). We reasoned that if GABAergic tone was reduced in KI flies, injection of PTX at subthreshold concentrations would trigger abnormal DLM spiking activity in *h^R^*^116^*^Q^* and *h^D^*^33^*^V^* but not *h^WT^*. Indeed, following injection of 30 µM PTX, none (zero of five) of the *h^WT^* flies displayed abnormal DLM spiking (Fig. 5 *G* and *H*). In contrast, two of five *h^R^*^116^*^Q^* and five of six *h^D^*^33^*^V^* flies displayed continuous DLM spiking following PTX injection (Fig. 5 *G* and *H*). In fact, we observed burst firing in *h^D^*^33^*^V^* individuals reminiscent of the spontaneous seizure phenotype in *h^D^*^33^*^V^* /*h^R^*^95^*^H^* flies, as revealed by similar looping trajectories in the ISI^–1^–CV_2_ plots (Fig. 5*I*). These experimental findings suggest thar a reduced GABAergic tone, perhaps arising from disrupted GABA synthesis, underlies the excitability and behavioral phenotypes seen in the *h^R^*^116^*^Q^* and *h^D^*^33^*^V^* flies.

### PLP Is Required for Both Development and Function of Adult Brain.

**Fig. 6.**
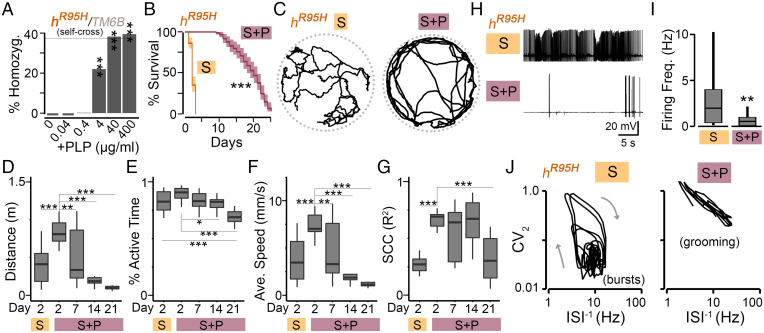
Development impairment, shortened life span, abnormal locomotion, and spontaneous seizure discharges in *h^R^*^95^*^H^* homozygotes are rescued by PLP supplementation. (*A*) Generation of *h^R^*^95^*^H^* homozygous flies from *h^R^*^95^*^H^*∕*TM*6*B* with PLP supplementation. The *χ*^2^ test of homogeneity compared with the control group with 0 µg/mL of PLP was used. *n* = 840 to 1,163. The dotted gray line indicates the homozygosity ratio (% Homozygo.) from *h^WT^/TM6B* (Fig. 3*A*). ***P < 0.001. (*B*) Life span of *h^R^*^95^*^H^* homozygotes (generated with 400 µg/mL of PLP) on sugar-only (S) or sugar supplemented with PLP (S + P; 400 µg/mL). *n* = 100 to 110. The log-rank test was used. ***P < 0.001. (*C*) Representative tracks during a 60-s interval of a single fly reared on S or S + P media for 2 d. (*D–G*) Quantification of *h^R^*^95^*^H^* walking over a 3-min interval for flies reared on indicated media. *n* = 9 to 16 flies. One-way ANOVA with Tukey’s post hoc was used. (*D*) Total distance traveled. (*E*) Percentage of active time. (*F*) Average speed. (*G*) SCC. (*H*) Representative traces of DLM firing from tethered *h^R^*^95^*^H^* flies. Note that the burst discharges present in flies reared on S media are suppressed by PLP supplementation. (*I*) Average firing frequency. *n* = 24 to 33. The two-tailed Student’s *t* test was used. *P < 0.05; **P < 0.01; ***P < 0.001. (*J*) Representative ISI^–1^–CV_2_ trajectories. Note that seizure-associated bursts are suppressed after PLP supplementation, and grooming (high-CV_2_) associated patterns are observed.

No *h^R^*^95^*^H^* homozygous flies were generated on the normal diet (Fig. 3*A*), suggesting that the PNPO activity in these homozygotes is insufficient to convert dietary VB6 to PLP to support development. Thus, PLP supplementation should rescue the developmental impairment. We further asked whether there is a basal level requirement for PLP to support development. To answer the question, we supplemented breeders with varying doses of PLP, ranging from 0 to 400 µg/mL. We found a dose-dependent rescue associated with PLP supplementation. Specifically, we observed a complete rescue with 40 or 400 µg/mL of PLP, a partial rescue with 4 µg/mL, and no rescue with doses below 4 µg/mL (Fig. 6*A*).

To examine whether PLP was also indispensable for adult flies, we maintained *h^R^*^95^*^H^* homozygous flies (developed with PLP supplementation; 400 µg/mL) on sugar with or without continued PLP supplementation (400 µg/mL). With PLP supplementation *h^R^*^95^*^H^* mutants survived for several weeks (Fig. 6*B*) (median life span: ∼18 d). In contrast, without PLP supplementation, these flies could not survive for more than 3 d, demonstrating that PLP is also required for the survival of adult flies.

Similar to *h^D^*^33^*^V^* /*h^R^*^95^*^H^* flies (Fig. 4), *h^R^*^95^*^H^* homozygous flies also exhibited seizure-like behaviors before their death. To characterize these abnormal behaviors and to further study the role of PLP in the behavioral output, we monitored the locomotor activity of *h^R^*^95^*^H^* homozygotes with or without PLP supplementation (Fig. 6*C* and Movie S3). Compared with *h^R^*^95^*^H^* homozygotes without PLP supplementation, *h^R^*^95^*^H^* homozygotes with PLP supplementation for 2 d showed significantly improved levels in total distance traveled, percentage of active time, average speed, and SCC (Fig. 6 *D*–*G*), demonstrating that PLP deficiency is responsible for the low behavioral performance of *h^R^*^95^*^H^* homozygous flies. The improvement of the behavioral performance declined with age, which correlates well with the increased lethality (Fig. 6*B*).

Consistent with our previous findings that PNPO deficiency leads to increased spontaneous firing and seizure discharges (27) (Fig. 5), *h^R^*^95^*^H^* homozygotes on sugar also exhibited clear spontaneous seizure discharges in the tethered fly preparation (Fig. 6*H* and Movie S4). Remarkably, these seizure-associated discharges were completely suppressed by PLP supplementation, as demonstrated by greatly reduced median firing rate (Fig. 6*I*) (1.9 to 0.5 Hz for flies on sugar and sugar only supplemented with PLP, respectively) and the alteration from burst-associated trajectories to grooming-associated patterns in the ISI^–1^–CV_2_ plots (Fig. 6*J*).

Taken together, results from stage-specific PLP supplementation demonstrate that PLP is required for both development and normal brain function in adult flies. Notably, PLP deficiency in larval stages will lead to a dose-dependent developmental impairment; the higher the level of a deficiency, the more severe the impairment.

### *h^WT^*/*h^R^*^95^*^H^* Flies Are Susceptible to Electroconvulsive Seizures Due to a Dominant-Negative Effect of h^R95H^.

**Fig. 7.**
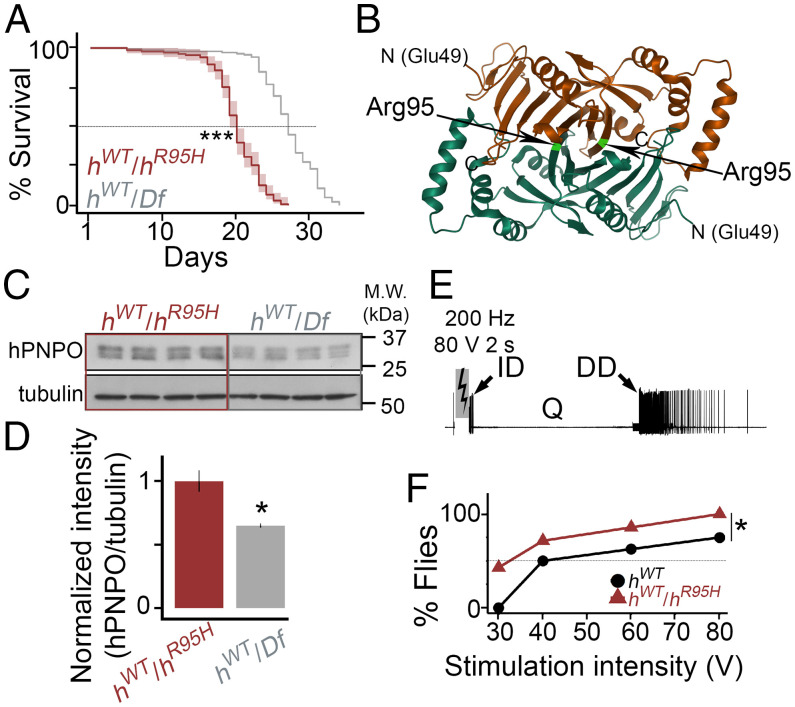
The *h^R^*^95^*^H^* allele has a dominant-negative effect on the *h^WT^* allele. (*A*) Survival of *h^WT^*/*h^R^*^95^*^H^* and *h^WT^*/*Df* flies on the sugar-only diet. *n* = 133 to 136. The log-rank test was used. ***P < 0.001. (*B*) The three-dimensional structure of dimerized PNPO (Protein Data Bank ID code 1NRG). The amino acid Arg95 is labeled on the structure. (*C*) Western blot of adult fly head homogenate from *h^WT^*/*h^R^*^95^*^H^* and *h^WT^*/*Df* flies. Tubulin is the loading control. *n* = 4 per genotype. M.W.: molecular weight. (*D*) Quantifications of the intensity of *hPNPO* bands from four biological replicates in each genotype. All three bands were included in the quantification. Error bars represent mean ± SEM. The two-tailed Student’s *t* test was used. *P < 0.05. (*E*) ECS activity. High-frequency stimulation across the brain (200 Hz for 2 s) can trigger a stereotypic seizure activity sequence: an initial spike discharge (ID), a quiescent period (Q), and a delayed spike discharge (DD). (*F*) The fraction of individuals displaying DD as a function of stimulation intensity. *n* = 6 to 8. Kruskal–Wallis nonparametric ANOVA was used. *P < 0.05.

We observed an unexpected lethality from *h^WT^*/*h^R^*^95^*^H^* flies when reared on sugar (Fig. 3*C*). To ascertain whether the lethality was due to haploinsufficiency or a dominant-negative effect of h^R95H ^on h^WT ^, we compared the survival rate of *h^WT^*/*h^R^*^95^*^H^* flies with that of *h^WT^*/*Df* flies in which the *sgll* gene is deleted in the *Df* line. Since there is only one *hPNPO* allele in *h^WT^*/*Df* flies, we reasoned that if the lethality was due to haploinsufficiency, survival of *h^WT^*/*h^R^*^95^*^H^* flies would be similar to *h^WT^*/*Df*. If the lethality was due to a dominant-negative effect, *h^WT^*/*h^R^*^95^*^H^* would exhibit worse survival than *h^WT^*/*Df* flies. As shown in Fig. 7*A*, the median survival rates are 19 and 27 d for *h^WT^*/*h^R^*^95^*^H^* and *h^WT^*/*Df*, respectively (*P* < 0.001), demonstrating a dominant-negative effect of h^R95H ^.

Functional PNPO is a dimerized protein (Fig. 7*B*) (14), so the dominant-negative effect of h^R95H ^on h^WT ^was likely mediated through the formation of a heterodimer between them. However, a reduced *hPNPO* protein level was observed in *h^R^*^95^*^H^*/*sgll^+^* flies (Fig. 2*A*), suggesting that the monomer or homodimer of h^R95H ^is not stable. How could the dominant-negative effect be possible if h^R95H ^is unstable? One possibility was that h^R95H ^was stabilized by forming a heterodimer with h^WT ^in *h^WT^*/*h^R^*^95^*^H^* flies. The stabilization was not observed in *h^R^*^95^*^H^*/*sgll^+^* flies because *h^R^*^95^*^H^* is less likely to form a heterodimer with SGLL due to their differences in the length and amino acid sequences (Fig. 1*C*). If h^R95H ^was indeed stabilized in *h^WT^*/*h^R^*^95^*^H^* flies, we would expect the hPNPO protein level in *h^WT^*/*h^R^*^95^*^H^* to be twice as high as that in *h^WT^*/*Df* since there are two alleles of *hPNPO* in *h^WT^*/*h^R^*^95^*^H^* and one in *h^WT^*/*Df*. That is exactly what we observed from western blots (Fig. 7 *C* and *D*). Thus, the dominant-negative effect of h^R95H ^is likely mediated through forming a heterodimer with h^WT ^.

The dominant-negative effect would presumably make *h^WT^*/*h^R^*^95^*^H^* flies susceptible to seizures. However, these flies did not show spontaneous seizures (Fig. 5 *A* and *B*). To determine whether *h^WT^*/*h^R^*^95^*^H^* flies were prone to seizures, we stimulated them with high-frequency electrical stimulation (HFS). HFS across the brain can induce stereotypic electroconvulsive seizure (ECS) discharges in flies (Fig. 7*E* and Movie S5) (40, 50). Compared with control flies, seizure-prone flies usually require lower stimulation intensities to induce seizures (25, 26). Many seizure-prone mutants have thus been identified and characterized (26, 37, 38, 40, 50, 51). By applying a range from 30 to 80 V of HFS intensities to *h^WT^*/*h^R^*^95^*^H^* heterozygous and *h^WT^* homozygous flies, we found a substantial reduction in the ECS stimulation threshold in *h^WT^*/*h^R^*^95^*^H^* compared with *h^WT^*. When *h^WT^*/*h^R^*^95^*^H^* and *h^WT^* flies were stimulated with a low voltage (30 V), seizures were already induced in 50% of *h^WT^*/*h^R^*^95^*^H^* flies, while no seizures were observed in *h^WT^* (Fig. 7*F*). The increased sensitivity in *h^WT^*/*h^R^*^95^*^H^* was maintained when the stimulation intensity was gradually increased. At 80 V, essentially all *h^WT^*/*h^R^*^95^*^H^* flies showed ECS discharges, while only about 60% of *h^WT^* were affected. Thus, the dominant-negative effect of *h^R^*^95^*^H^* confers the susceptibility of *h^WT^*/*h^R^*^95^*^H^* flies to HFS-induced seizures.

## Discussion

Studying metabolic genes presents unique opportunities to uncover the contribution of gene–environment interactions to human health conditions and can offer potential treatment options for diseases. Yet, it is challenging to study mutations of such genes in humans since they often lead to lethality during development or rare diseases due to the functional essentiality of metabolic genes. Here, we generated a series of KI *Drosophila* models carrying *hPNPO* variants and examined these epilepsy-associated variants at molecular, circuitry, behavioral, and organismal levels. This genetically tractable model system and well-controlled dietary conditions have enabled the functional and molecular characterization of three epilepsy-associated *PNPO* alleles with vastly different phenotypes. Our results reveal that *PNPO* has multiple biological functions; that different *PNPO* variants have distinct molecular properties; and that individual *PNPO* mutations, diet, and allele–diet interactions all contribute to the final phenotype expression.

Our data demonstrate how the diet’s effect on the phenotype expression depends on *PNPO* alleles, with more evident outcome in genotypes showing milder deficiency of PNPO activity. For example, with specified dietary breeding and testing conditions, *h^R^*^116^*^Q^*, *h^D^*^33^*^V^* , or *h^WT^*/*h^R^*^95^*^H^* flies exhibited readily detectable behavioral outcomes, whereas *h^D^*^33^*^V^* /*h^R^*^95^*^H^* stayed relatively invariant (Fig. 4 and *SI Appendix*, Fig. S8). Notably, a less variable phenotype is also known for patients carrying severe PNPO mutations. The seizure onsets in patients with severe PNPO mutations (e.g., R95H) range from hours to weeks compared with the wide variation spanning from hours to years in patients with mild mutations (e.g., R116Q) (16). The less profound effect of diet in KI flies or patients with severe PNPO deficiency is presumably due to the diminished capability of mutant PNPOs in converting dietary VB6 to PLP.

On the other hand, although mild PNPO mutants retain a capacity to convert dietary VB6 to PLP to a greater extent and it is less likely for them to cause acute deleterious effects, the fact that *h^R^*^116^*^Q^* flies exhibited a shortened life span on the normal diet (Fig. 3*B*) suggests that the deleterious effect of mild PNPO deficiency can be cumulative. While R95H and D33V mutations are rare in the general population, R116Q is relatively common; ∼10% are carriers, and 1% are homozygous (52). Therefore, it will be important to examine whether mild PNPO deficiency caused by R116Q can be exacerbated by other genetic and/or environmental factors to cause epilepsy or other diseases in humans.

Developmental delay is one common symptom manifested by PNPO-deficient patients (3, 17, 19, 53), raising the question of whether specific developmental impairments lead to seizures. By providing flies with PLP during either the larval or adult stages, we have demonstrated that developmental impairments and seizures in adults are dissociable (Fig. 6). Our data also indicate that prenatal PLP supplementation may be beneficial for the development of fetuses who carry severe PNPO mutations.

A major finding from this study is the dominant-negative effect of the R95H mutation. Neonatal epileptic encephalopathy caused by PNPO deficiency is generally thought to be an autosomal recessive disease (Online Mendelian Inheritance in Man no. 610090), implying that PNPO mutant carriers do not show any overt phenotype. Indeed, in all reported PNPO deficiency cases, 78% are homozygous for a specific mutation, and 21% are compound heterozygotes (16), except for one patient reported to be a heterozygote (3). Yet, in KI flies, we found a readily detectable dominant-negative effect from the *h^R^*^95^*^H^* allele (Fig. 7). The dominant-negative effect is presumably associated with heterodimer formation between h^R95H ^and h^WT ^. Structural studies have shown that amino acid R95 (arginine 95) is required for the cofactor FMN binding (14, 54) and thus, PNPO enzyme activity (14). An altered FMN binding site may lead to global conformation changes and hence, a partially or fully inactive enzyme. It is tempting to predict that mutations that do not impede dimerization but affect FMN binding can have a dominant-negative effect. Therefore, human carriers of such a category of PNPO mutations could be susceptible to epilepsy and related disorders.

We find a unique pattern of spontaneous DLM burst firings in some *h^D^*^33^*^V^* flies. The individual variability could be due to many factors, including stochastic events in the developmental processes into adults (Fig. 3*A*). Notably, distinct electroencephalogram (EEG) patterns have been reported in patients carrying the D33V mutation, who often exhibit a unique pattern of temporal sharp waves or multifocal sharp waves instead of the burst suppression in the majority of PNPO-deficient patients (55). The molecular and neural mechanisms associated with this peculiar mutation underlying unique firing or EEG pattern remain to be elucidated in both *Drosophila* and humans. The amino acid D33 (aspartate 33) is located in the N terminus of the PNPO protein, and the N terminus of PNPO is often not retained in crystal samples (Fig. 7*B*) (14); thus, it is often challenging to examine D33V in structural studies. Our molecular studies on *h^D^*^33^*^V^* KI flies show a decrease in the mRNA level and consequently, the protein level of PNPO (Fig. 2 *A–C*). However, it seems unlikely that the decreased PNPO protein level is a major determining factor for the distinct firing in *h^D^*^33^*^V^* flies since a decreased PNPO protein level was also observed in *h^R^*^95^*^H^* flies (Fig. 2*A*), which exhibit only regular seizure discharges (Fig. 6 *H–J*). It is worth noting that *h^R^*^95^*^H^* flies have a higher level of protein decrease and a greater level of enzymatic activity loss than *h^D^*^33^*^V^* flies; thus, there is a possibility that firing patterns are associated with the degree of PNPO deficiency. Future studies should investigate whether the D33V mutant protein alters neuronal or circuit functions in unique ways and whether different extents of PNPO deficiency can affect the various categories of neural function in a severity-dependent manner.

The molecular and functional consequences of R116Q have been controversial. R116Q was first reported in patients with neonatal epileptic encephalopathy (3), suggesting that it is likely a severe mutation. Later clinical reports show that unlike seizures in patients carrying other PNPO mutations, seizures in R116Q patients can occur beyond the neonatal stage (3–5), indicating that R116Q is more likely to be a relatively mild mutation. The molecular consequence of R116Q is also inconclusive. An earlier in vitro study showed that R116Q decreases the enzyme activity to 83% of the WT protein (3). In contrast, two recent studies report decreases to ∼40 and ∼1% of the WT activity instead (4, 56). Our in vivo studies indicate that R116Q is likely a mild mutation (Figs. 3*A* and 5 *A* and *B*) and raise the possibility that genetic background or environmental factors can contribute to the above variability in data derived from R116Q patients (3). However, despite mild behavioral phenotype, at the cellular level, R116Q profoundly increases the terminal localization of hPNPO protein in the fly CNS (Fig. 2 *D* and *E*). Whether this altered localization of h^R116Q ^has a subtle effect on neuronal function is unknown.

The neural mechanisms underlying PNPO deficiency-induced seizures remain to be determined. Our previous studies on spike pattern analyses from *sgll^95^* and *sgll* knockdown flies have suggested that GABA dysfunction contributes to PNPO deficiency-induced seizures (27). In this study, we show that *h^D^*^33^*^V^* flies are more vulnerable to GABA_A _receptor blockade (Fig. 5*G*), suggesting that these flies may have a reduced GABAergic tone, presumably arising from decreased GABA synthesis. In agreement with these results, reduced GABA levels have been recently reported in a zebrafish *PNPO* knockout model (57). Therefore, PNPO deficiency probably leads to a reduced GABAergic tone, which decreases the firing threshold and/or promotes synchronous firing.

It should be noted that PLP exhibits considerable functional complexities that can complicate the inference of the neurotransmitter system responsible for the seizures and may account for the variable biochemical measurements among PNPO-deficient patients (17). In addition to being a cofactor and involved in the synthesis of several neurotransmitters, PLP has other cellular functions that can potentially modify the eventual expression of seizures, including altering immune function and acting as an adenosine triphosphate antagonist at P2 purinoceptors (58). One interesting question is why seizures in PNPO-deficient patients generally do not respond to available antiepileptic drugs, including those targeting GABA signaling (59). One possibility is that GABA dysfunction leads to initiation of seizure-prone conditions, which may be functionally compensated by subsequent remodeling of neuron circuitry and modified involvement of other neurotransmitters, such as dopamine and serotonin. This notion is partially supported by the fact that some PNPO-deficient patients show initial responses to GABA-related drugs (5, 18, 19, 60), although seizures in them ultimately become drug resistant. Thus, future investigations into the complexity of neuronal network dynamics, the role of various PLP-dependent neurotransmitters, and other interactive biological functions of PLP are required to elucidate the process in generating PNPO deficiency-induced seizures.

In summary, our studies uncover a diversity of molecular defects of *PNPO* variants, reveal the role of the *PNPO* allele–diet interaction in the phenotype expression, and highlight the contribution of PNPO deficiency to epilepsy in general. These data provide biological bases for understanding phenotypic variations in PNPO deficiency patients and have significant clinical implications in developing treatment strategies. These studies also demonstrate that KI *Drosophila* models are valuable for systematically analyzing the functional and molecular effects associated with each *PNPO* allele identified in epilepsy patients.

## Materials and Methods

### Generation of KI Strains.

Four different KI strains were generated using CRISPR-Cas9 technology (61). The WT *hPNPO* cDNA was amplified from the human brain cDNA library (TaKaRa; catalog no. 637242) (27). Three mutations were introduced separately by mutagenesis. The single guide RNAs, Cas9 mRNAs, and donor constructs were injected into embryos from flies with a genotype of *w^1118^/FM7a; Bc/CyO; TM3/TM6B, Hu, Tb* (http://www.fungene.tech/) (*SI Appendix*).

### *Drosophila* Husbandry.

Flies were generated on either standard CYM or FB media (34). Flies used in all experiments were raised and tested at room temperature (∼23 ^∘^C) in a 12:12-h light:dark cycle (*SI Appendix*).

### Western Blotting.

Total protein from adult heads was extracted, loaded for sodium dodecyl sulfate–polyacrylamide gel electrophoresis, and detected with rabbit anti-hPNPO and mouse anti–*β*-tubulin antibodies (*SI Appendix*).

### qRT-PCR.

Total RNA was extracted and reversely transcribed to cDNA for qPCR. Two pairs of primers were designed to target the *hPNPO* KI alleles (N- and C-terminal regions, respectively). A pair of primers was used to amplify *rp49* (*SI Appendix*).

### Immunohistochemistry.

The protocol was adapted from the Flylight protocol (62). Briefly, brains were dissected, fixed, and stained with rabbit anti-hPNPO and mouse anti-Brp antibodies. Signals for hPNPO were amplified with the Tyramide SuperBoost kit (Invitrogen; catalog no. B40926). DAPI was added into the wash buffer to stain the nucleus when needed. Images were taken using a Leica SP5-II-STED-CW confocal microscope and processed in Fiji (63) (*SI Appendix*).

### Developmental Assay.

A cohort of 2 to 3 flies per sex was set up in a single vial, or a group of 12 to 15 flies per sex was set up in a single bottle. Flies eclosed within 6 d from each cross were examined for the Balancer marker (*SI Appendix*).

### Life Span and Survival Study.

Fifteen to 20 male flies were maintained in vials filled with the standard CYM medium (the normal diet condition) or 4% sucrose in 1% agar (the sugar-only condition). Daily survival was recorded (*SI Appendix*).

### Behavioral Recording.

Single fly or multiple flies were recorded and tracked using IowaFLI Tracker (27, 64). The total distance traveled, average speed, percentage of active time, and SCC were further calculated as previously described (27) (*SI Appendix*).

### Electrophysiology.

Action potentials were recorded from DLM muscles as previously described (27, 65). Drugs were delivered via dorsal vessel injection (41, 66), and GF stimulation was delivered through tungsten electrodes inserted in each cornea as previously described (67). ECS discharges were induced by HFS across the brain delivered by the same electrodes (40, 65). The stimulation protocol consisted of a 2-s train of 0.1-ms pulses delivered at 200 Hz at a specified voltage (30 to 80 V) (*SI Appendix*).

### Statistical Analysis.

Statistical analysis was performed in MATLAB (R2019b) or R (version 3.6.1). Details on statistical analyses, including sample sizes, tests performed, and multiple test correction if necessary, are provided in the figures.

## Supplementary Material

Supplementary File

Supplementary File

Supplementary File

Supplementary File

Supplementary File

Supplementary File

## Data Availability

Custom scripts have been deposited in GitHub (https://github.com/IyengarAtulya/IowaFLI_tracker and https://github.com/IyengarAtulya/SpikeTrainAnalysisToolkit). All study data are included in the article and/or supporting information.
