# Supplementary material for: Drosophila carrying epilepsy-associated variants in the vitamin B6 metabolism gene PNPO display allele- and diet-dependent phenotypes
Source: Proc Natl Acad Sci U S A. 2022 Feb 25;119(9):e2115524119. doi: 10.1073/pnas.2115524119 (PMC8892510; doi:10.1073/pnas.2115524119)
Supplement: Supplementary File [file pnas.2115524119.sapp.pdf]

PNAS
www.pnas.org

Supplementary Information for

Drosophila carrying epilepsy-associated variants in the vitamin B6 metabolism gene *PNPO* display allele- and diet-dependent phenotypes

Wanhao Chi, Atulya SR Iyengar, Wenqin Fu, Wei Liu, Abigayle E Berg, Chun-Fang Wu, Xiaoxi Zhuang

Correspondence should be addressed to: xzhuang@bsd.uchicago.edu or chun-fang-wu@uiowa.edu

This PDF file includes:

Supplementary text
Figs. S1 to S8
Tables S1 to S2
Legends for Movies S1 to S5
SI References

Other supplementary materials for this manuscript include the following:

Movies S1 to S5

Supporting Information Text

Materials and Methods.

Generation of knock-in strains. Four different KI strains were generated using CRISPR/Cas9 technology (1).The *Drosophila PNPO* gene (*sugarlethal*) (2) was replaced by either WT human *PNPO* (*hPNPO*) cDNA or one of three mutant *hPNPO* cDNAs. The WT *hPNPO* cDNA was amplified from the human brain cDNA library (TaKaRa, Cat no. 637242) (3). The c.98A > T mutation (corresponding to p.D33V), c.284G > A mutation (corresponding to p.R95H), and c.347G > A mutation (corresponding to p.R116Q) were introduced separately by mutagenesis. The single guide RNAs (sgRNAs) were designed with CRISPR Optimal Target Finder (4) and transcribed in vitro as described in the published protocol (5) (*SI Appendix*, Table S1). Cas9 messenger RNAs (mRNAs) were transcribed in vitro from plasmid MLM3613 (Addgene, plasmid no. 42251). Donors were various *hPNPO* cDNAs assembled in the pBluescript SK(-) vector. The sgRNAs, Cas9 mRNAs, and donor constructs were injected into embryos from flies with a genotype of w^{1118}*/FM7a; Bc/CyO; TM3/TM6B, Hu, Tb* (http://www.fungene.tech/). The introduction of the mutation in KI alleles was confirmed by Sanger sequencing of PCR products amplified with a pair of PCR primers that specifically targeted *hPNPO* cDNA (*SI Appendix*, Table S1).

The mutant alleles were initially maintained in heterozygotes with the *TM6B, Hu, Tb* balancer, but KI homozygotes gradually took over heterozygotes in h^{WT} and h^{R116Q} breeding bottles.

***Drosophila* husbandry.** Electrophysiology experiments and some behavioral recordings (indicated below) were performed on flies bred on Frankel & Brosseau's (FB) media (6) at the University of Iowa. For all other experiments, flies were generated on standard Cornmeal-Yeast-Molasses (CYM) media from the Fly Kitchen at the University of Chicago. Flies used in all experiments were raised and tested at room temperature ($\sim$ 23 ºC) in a 12:12-h light:dark cycle. The deficiency lines *Df(3R)BSC221/TM6B* and *Df(3R)ED5223/TM6C* were obtained from the Bloomington Drosophila Stock Center (BDSC no. 9698 and 9076).

Western blotting. Male flies, 1- to 2-d old, were used. Total protein from 30 male adult heads was extracted and quantified. A total of 50 μg protein from each sample was loaded for SDS-PAGE. Separated proteins were electrophorectically transferred to the PVDF membrane. After blocking, the membrane was incubated with primary antibodies and then the secondary antibodies (*SI Appendix*, Table S2). Signals were detected with chemiluminescence (ThermoScientific, Cat no. 32109).

RNA extraction, reverse transcription, and quantitative PCR. Total RNA was extracted from 30 male adult heads using the RNA extraction kit (Zymo Research, Cat no. R2030). After removal of genomic DNA using the DNA-free kit (AMBION, Cat no. M1906), total RNA was used for reverse transcription using the SMARTScribe Reverse Transcription Kit (TaKaRa, Cat no. 639537).

Quantitative PCR (qPCR) was performed using SYBR Green real-time PCR method (Applied biosystems). The Ct values were defined by the default settings in QuantStudio3 (Thermofisher) using a run method of 2 min at 95 °C followed by 40 cycles of 15 s at 95 °C and 1 min at 60 °C. Relative gene expression was calculated as $2^{\triangle\triangle \mathrm{Ct}}$, where $\triangle$Ct was determined by subtracting the average Ct value from the housekeeping gene *ribosomal protein 49* (*rp49*) and $\triangle\triangle$Ct was the difference of the $\triangle$Ct between the corresponding genotype and the reference genotype.

Two pairs of primers were designed to target the *hPNPO* KI alleles (N- and C- terminal regions, respectively). A pair of primers were used to amplify *rp49*. Primers were pre-tested for single-product amplification. Primer sequences are shown in *SI Appendix* Table S1.

Immunohistochemistry staining and confocal imaging. The protocol was adapted from the Flylight protocol (7). Briefly, brains from 1- to 2-d old flies were dissected in cold S2 media and fixed with 2% paraformaldehyde for 55 min. After a brief rinse with 0.5% Triton X-100, brains were blocked with blocking solution (5% normal donkey serum and 0.5% TritonX-100 in phosphate-buffered saline) for 1.5 h and then were incubated with primary antibodies (*SI Appendix* Table S2). Signals for hPNPO were amplified with the Tyramide SuperBoost kit (Invitrogen, Cat no. B40926) by following the manufacturer's protocol. After incubation with the secondary antibodies and washes, brains were mounted on a double-frosted slide and covered with coverslips. DAPI was added into the wash buffer to stain the nucleus when needed. Images were taken using Leica SP5-II-STED-CW confocal microscope in the Integrated Light Microscopy Core Facility at the University of Chicago and processed in Fiji (8).

Developmental assay with or without PLP supplementation. Male and female heterozygous flies were bred in normal food vials or bottles with or without PLP supplementation. A cohort of 2 to 3 flies per sex was set up in a single vial, or a group of 12 to 15 flies per sex was set up in a single bottle. F1 flies eclosed within 6 d from each cross were examined for the Balancer marker.

Complementation test. Female flies, either homozygous (h^{WT}, h^{R116Q}, and h^{D33V}) or heterozygous (h^{R95H}/*TM6B*), and male flies from one of the *Df* strains were picked for breeding. A cohort of 2 to 3 such flies per sex was set up for each combination. F1 flies eclosed within 6 d from each cross were examined for the Balancer marker.

Lifespan and survival study. Fifteen to 20 male flies, 1- to 2-d old, were maintained in vials filled with the standard CYM medium (the normal diet condition) or 4% sucrose in 1% agar (the sugar-only condition). When there was PLP supplementation, PLP was added to the vial to the final concentration of 400 μg/mL. Flies were transferred to new vials one to two times a week. Daily survival was recorded.

Single- or multi-fly behavioral recording. Male flies, 1- to 2-d old, were reared on either control diet, sugar-only diet, or sugar + 400 μg/mL PLP diet for 4 to 6 d. For single-fly recordings, one fly was loaded without anesthesia into a circular arena (diameter = 60 mm) prefilled with 1% agar and was recorded with a camera (FLIR Flea 3) for 3 min at 20 fps (frames per second) (Fig. 4 *A-E*). For multi-fly recordings, 4 flies were loaded without anesthesia into a circular arena (diameter = 28 mm) seated on filter paper (Whatman no. 1). Flies were recorded with either a webcam (Logitech c920, Fig. 4 *F-J*, 10 min at 30 fps), or a camera (Flea3, Fig. 6 *C-G*, 3 min at 20 fps). After recording, fly positions in each frame were automatically tracked using IowaFLI Tracker (3, 9). The total distance travelled, average speed, percentage of active time, and speed correlation coefficient were further calculated as described previously (3).

Tethered fly electrophysiology and data analysis. Tethered fly electrophysiological experiments were performed as described previously (3, 10). Briefly, flies were immobilized on ice, affixed onto a tungsten wire between the head and thorax, and allowed 30 min for recovery. Flight muscle spikes were recorded by an electrolytically sharpened tungsten electrode inserted into the dorsal longitudinal muscle (DLMa). A sharpened tungsten electrode inserted into the dorsal abdomen served as a reference. Signals were amplified by a Model 1800 AC amplifier (AM systems) and were digitized by a USB 6212 data acquisition card (National Instruments) controlled by a custom LabVIEW 2018 script. Spikes were identified offline by a custom MATLAB script.

The average firing rate was determined by dividing the total spike count during a recording session by the duration ($\sim$ 90 s). The instantaneous firing frequency was defined as the reciprocal of an inter-spike interval (i.e., ISI^{-1}). The instantaneous coefficient of variation (CV_2), a measure of firing regularity, was defined between two adjacent inter-spike intervals, *i* and *i+1*, as: $2|ISI^{-1}{}_{i} - ISI^{-1}{}_{i+1}|/(ISI^{-1}{}_{i} + ISI^{-1}{}_{i+1}|)$ (11). Lower CV_2 values indicate with rhythmic spiking, while high CV_2 values correspond with irregular spiking. Plots of the ISI^{-1} versus CV_2 trajectories for a spike train were computed as described previously (12).

Systemic application of nicotinic acetylcholine receptor blocker mecamylamine and the $GABA_A$ blocker picrotoxin (PTX) was performed via a dorsal vessel injection procedure (12, 13). Briefly, mecamylamine (Sigma, Cat no. M9020) or PTX (Sigma, Cat no. P1675) was dissolved in adult hemolymph-like solution (14) to a final concentration (100 μM or 30 μM, respectively) and marked with blue no. 1 dye (16 mg/mL). A filamented glass microelectrode was filled with 0.33 of μl solution and inserted into the dorsal vessel. Positive air pressure pushed the solution into the dorsal vessel which systemically circulated the drug within seconds.

Giant fiber stimulation was delivered through tungsten electrodes inserted in each cornea as previously described (15). Stimuli consisted of brief pulses (0.1 ms duration, 30 V amplitude), which reliably activated the GF neuron and downstream elements. Electroconvulsive seizure discharges were induced by high-frequency electrical stimulation across the brain delivered by the same electrodes (10, 16). The stimulation protocol consisted of a 2-s train of 0.1 ms pulses delivered at 200 Hz at a specified voltage (30 - 80 V).

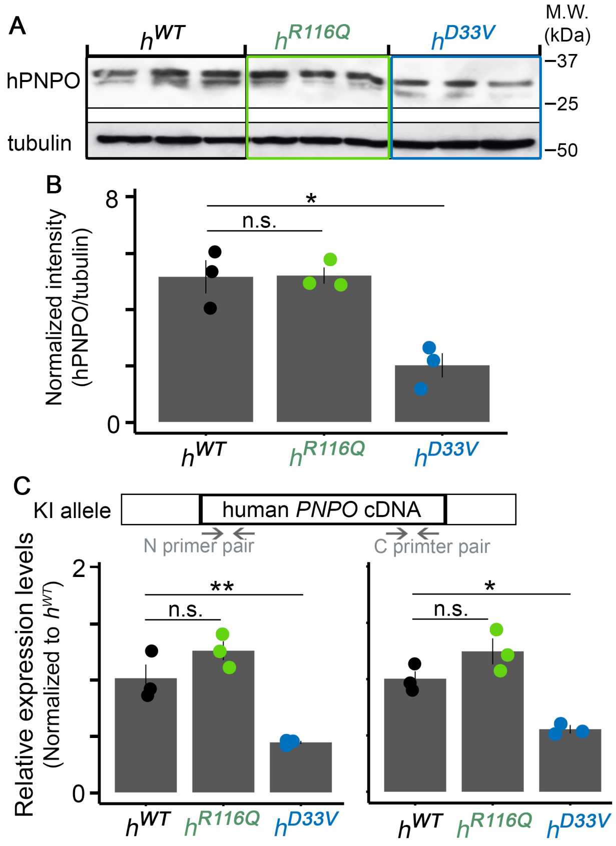

Fig. S1. The *hPNPO* mRNA and protein levels in KI homozygotes reared on the normal food. (*A*) Western blot of adult fly head homogenate from h^{WT}, h^{R116Q}, and h^{D33V} homozygotes. n = 3 per genotype. Tubulin is the loading control. (*B*) Quantifications of hPNPO protein level in *A* (all bands). (*C*) Expression of *hPNPO* at the mRNA level. n = 3 per genotype. Error bars represent Mean ± SEM. n.s. $P > 0.05$, * $P < 0.05$, ** $P < 0.01$. Two-tailed student's *t*-test with Bonferroni's correction.

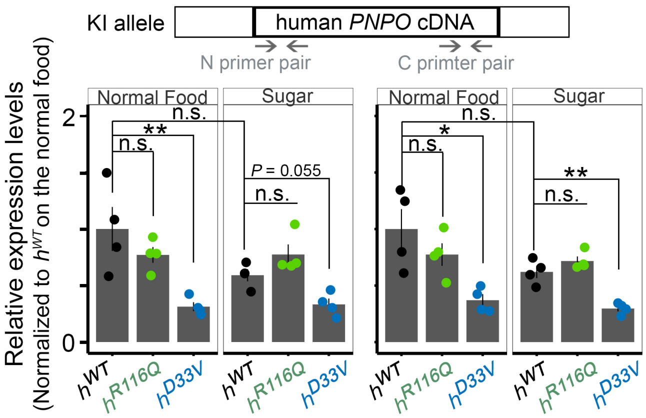

Fig. S2. The *hPNPO* mRNA levels in KI homozygotes reared on the normal or sugar-only diet. n = 4 per genotype per condition. Error bars represent Mean $\pm$ SEM. n.s. $P > 0.05$, * $P < 0.05$, ** $P < 0.01$. Two-way ANOVA was performed to examine the genotype by food condition interaction. The P values for the interaction term equal to 0.071 and 0.164 for the N- and C-primer pair, respectively. One-way ANOVA with Tukey's *post-hoc* was performed to examine the genotype's effect under each food condition. Two-tailed student's t-test was performed to examine the food's effect on the *hPNPO* level in h^{WT}.

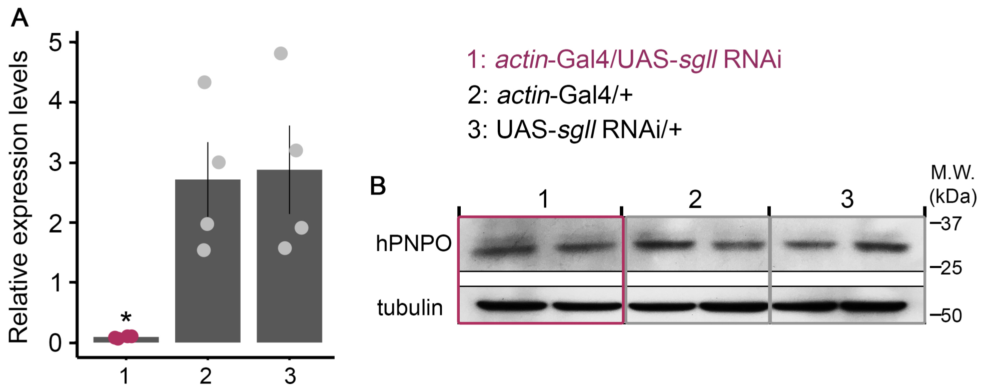

Fig. S3. The antibody used for detecting hPNPO does not seem to recognize *Drosophila* PNPO. (*A*) The *sgll* mRNA levels in ubiquitous *sgll* knockdown (genotype: *actin*-Gal4/UAS-*sgll* RNAi) and control flies (genotypes: *actin*-Gal4/+ and UAS-*sgll* RNAi/+). n = 4 per genotype. Error bars represent Mean $\pm$ SEM. * $P < 0.05$. One-way ANOVA with Tukey's post hoc. (*B*) Western blots from adult head homogenates with various genotypes. n = 2 per genotype. Tubulin was the loading control. One bind was detected from all three genotypes. The size of this band seems to be correct; the predicted molecular weight for *Drosophila* PNPO ($\sim$ 27 KDa). However, the band intensity in *sgll* knockdown flies is as same as that in two controls, indicating that this band is less likely to be *Drosophila* PNPO.

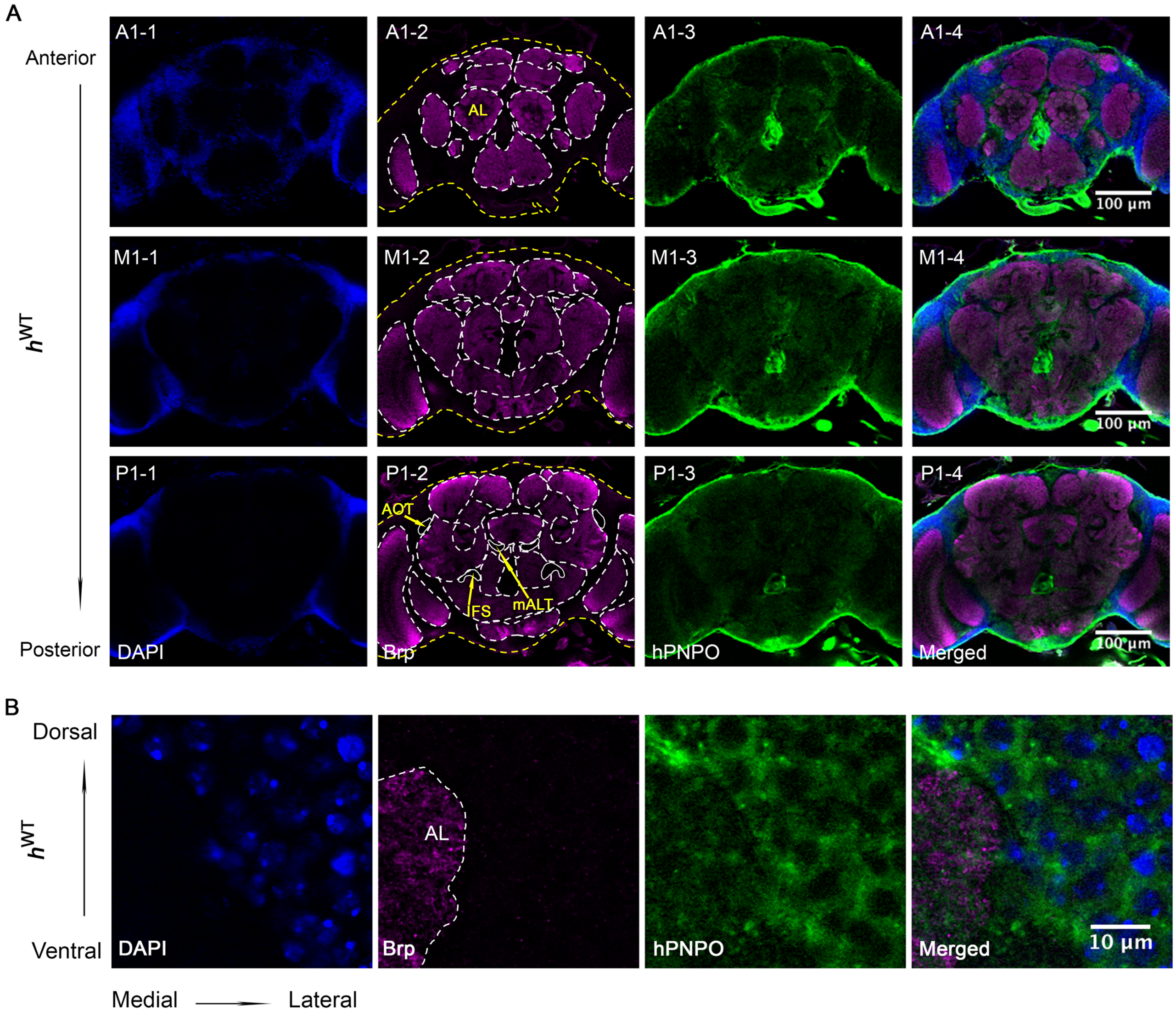

Fig. S4. Expression pattern of h^{WT} in the adult brain. (*A*) h^{WT} was ubiquitously expressed in the brain with the strongest staining from the cell body rind, a structure equivalent to the cortex in mammals (17). Relatively strong staining was also observed in the areas surrounding neuropils, e.g., antennal lobe (AL). There was little overlap between Brp and hPNPO staining, suggesting that h^{WT} is not enriched in the terminal structure. Consistently, no staining was observed in fiber bundles, such as anterior optic tract (AOT), inferior fiber system (IFS), and medial antennal lobe tract (mALT). (*B*) hPNPO staining does not overlap with DAPI, a nucleus marker, suggesting that hPNPO is a cytosolic protein. Blue, DAPI; Magenta, Brp; Green, hPNPO.

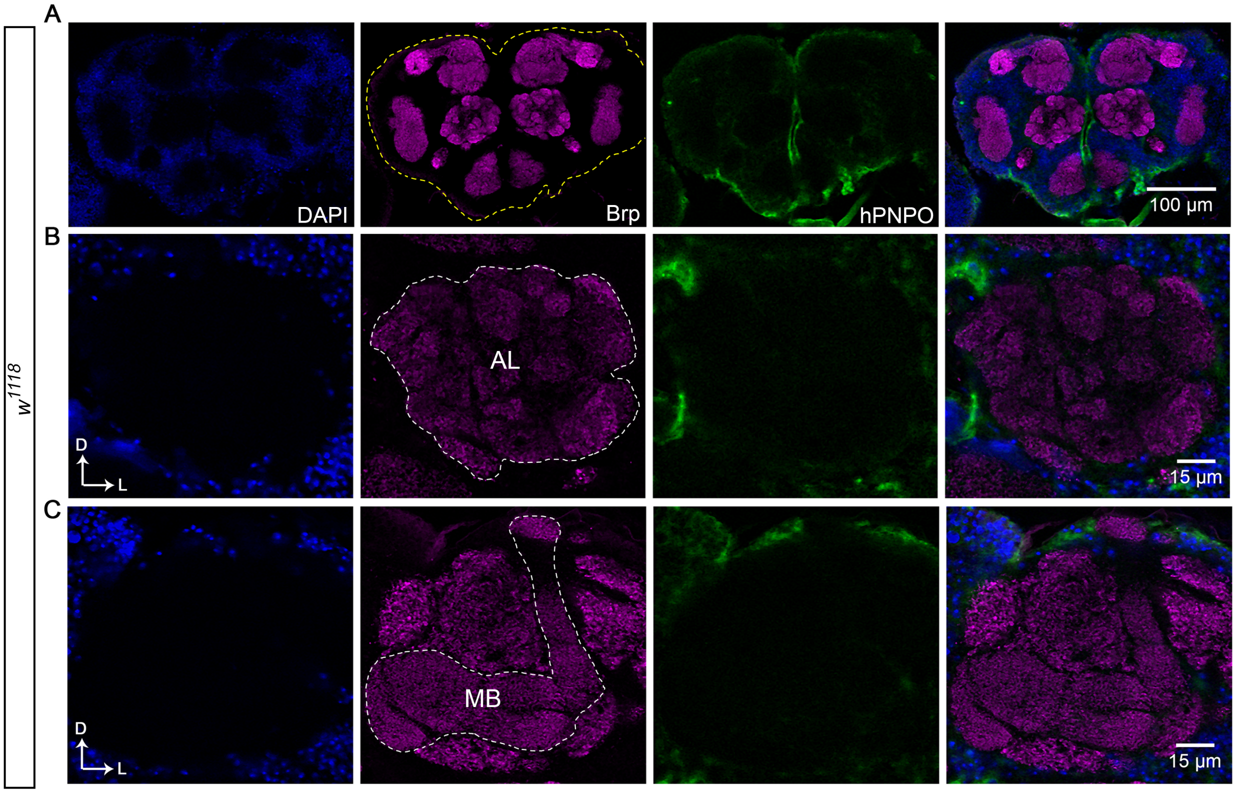

Fig. S5. hPNPO staining in w^{1118} control flies. (*A*) Weak hPNPO staining in the cell body rind. (*B-C*) Little or no hPNPO staining in the antennal lobe (AL) and mushroom body (MB).

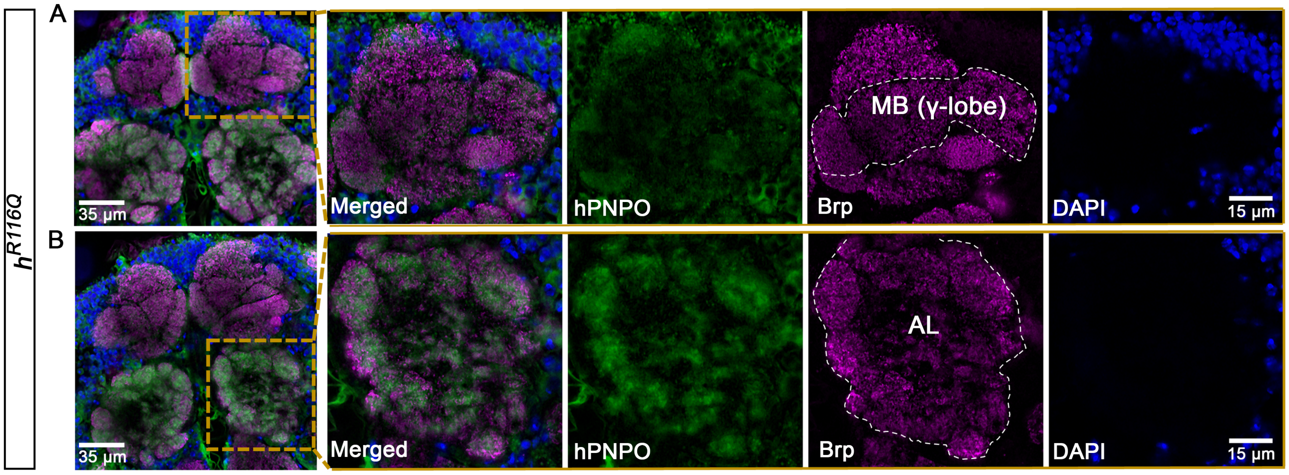

Fig. S6. hPNPO staining in the h^{R116Q} brain. (*A*) hPNPO staining in the mushroom body (MB). (*B*) hPNPO staining in the antennal lobe (AL). Compared to AL, MB has faint hPNPO staining.

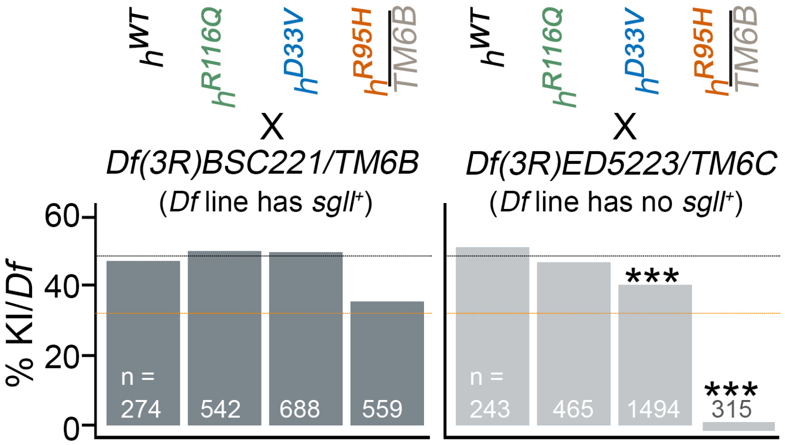

Fig. S7. Complementation test. Two *Deficiency* (*Df*) lines were used: one experimental line (genotype: *Df(3R)ED5223TM6C,Sb*) with the *sgll* gene deleted and one control line (genotype: *Df(3R)BSC221TM6B,Tb*) with a chromosome deletion adjacent to that of the experimental line. Each *Df* line was individually crossed with either homozygous flies from h^{WT}, h^{R116Q}, and h^{D33V} lines or heterozygous flies from h^{R95H} (due to homozygous lethality). Two dotted lines represent the expected ratio of KI/*Df* from breeding with three homozygous lines and the h^{R95H}/*TM6B* line, respectively. *** $P < 0.001$, The χ^2 test of homogeneity compared with the expected value.

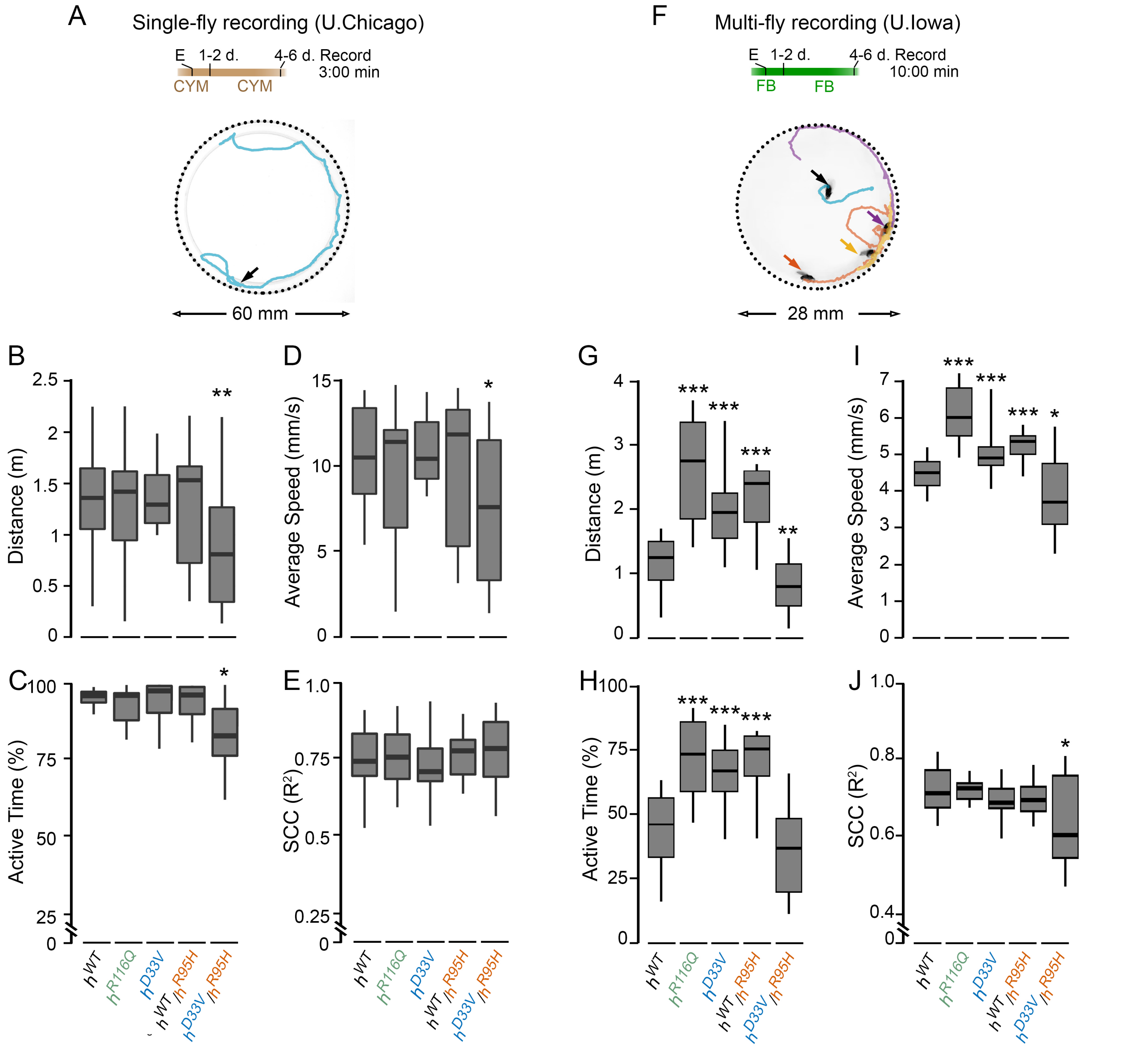

Fig. S8. Behavioral analyses of KI flies on the standard diets. (*A*, *F*) Breeding and testing conditions. Flies were eclosed (E) and maintained on either CYM or FB diet. (*B-E*) Total travelled distance, average speed, percentage of active time, and speed correlation coefficient (SCC) from flies in condition A. n = 18-24. (*G-J*) Total travelled distance, average speed, percentage of active time, and speed correlation coefficient (SCC) from flies in condition F. n = 32-40. * $P < 0.05$, ** $P < 0.01$, ** $P < 0.001$. Two-tailed student's *t*-test with Bonferroni's correction compared with h^{WT}.

Table S1. gRNA and PCR primer sequences

Generation of KI flies	gRNA-1-1	GTGGCACCTCCGAGGCACCTTGG
	gRNA-1-2	GTTCCACAGAGTTACCAGCAGG
	gRNA-2-1	GAACCAGTTTGCTGTCGACTTCGG
	gRNA-2-2	GGTTGGGTGTACGAACGGCTGG
KI mutation confirmation	Primer Forward	TGAAGTTGCTGCAAACAATTCGAAGG
	Primer Reverse	CTAAGGTGCAAGTCTCTCATAGAGCC
qPCR primers	KI-N Forward	ATGACGTGCTGGCTGCG
	KI-N Reverse	ACCACACAGGTGACTGAGGTA
	KI-C Forward	CTCAGGTGATGGAGTTCTGGCA
	KI-C Reverse	CTAAGGTGCAAGTCTCTCATAGAGCC
	rp49 Forward	GCTAAGCTGTCGCACAAATG
	rp49 Reverse	GTTCGATCCGTAACCGATGT

Table S2. Antibodies for Western blotting (WB) and Immunohistochemistry (IHC) staining

Antibody	Source	Cat #	Dilution
Rabbit anti-human PNPO	Novus	NBP1-87302	1:500 (WB); 1:300 (IHC)
Mouse anti-β-tubulin	DSHB	E7	1:500 (WB)
Goat anti-rabbit HRP	Jackson ImmunoResearch	111-035-144	1:10,000 (WB)
Goat anti-mouse HRP	Jackson ImmunoResearch	115-035-003	1:10,000 (WB)
Mouse anti-Brp	DSHB	nc-82	1:10 (IHC)
Flurophore conjugated Donkey anti-primary antibody species	Life Technologies	A21206; A21202; A31573; A31571	1:400 (IHC)
Flurophore conjugated Donkey anti-primary antibody species	Jackson ImmunoResearch	715-585-150; 711-585-152	1:400 (IHC)

Legends for the video are below:

Movie S1. Representative video recordings of KI flies

Movie S2. Representative DLM recordings of KI flies

Movie S3. Representative video recordings of h^{R95H} flies with or without PLP supplementation

Movie S4. Representative DLM recordings of h^{R95H} flies with or without PLP supplementation

Movie S5. Representative DLM recordings of h^{WT} flies upon high-frequency electrical stimulation

References

1. X Zhang, WH Koolhaas, F Schnorrer, A versatile two-step crispr- and rmce-based strategy for efficient genome engineering in drosophila. *G3: Genes, Genomes, Genet.* (2014).
2. W Chi, L Zhang, W Du, X Zhuang, A nutritional conditional lethal mutant due to pyridoxine 5'-phosphate oxidase deficiency in drosophila melanogaster. *G3; Genes,Genomes,Genetics* **4**, 1147–1154 (2014).
3. W Chi, et al., Pyridox (am) ine 5'-phosphate oxidase deficiency induces seizures in drosophila melanogaster. *Hum. Mol. Genet.* **28**, 3126–3136 (2019).
4. SJ Gratz, et al., Highly specific and efficient crispr/cas9-catalyzed homology-directed repair in drosophila. *Genetics* **196**, 961–971 (2014).
5. AR Bassett, C Tibbit, CP Ponting, JL Liu, Highly efficient targeted mutagenesis of drosophila with the crispr/cas9 system. *Cell reports* **4**, 220–8 (2013).
6. A Frankel, J G.E.Brosseau, A drosophila medium that does not require dried yeast. *Drosophila. Inf. Serv.* **43**, 184 (1968).
7. BD Pfeiffer, et al., Tools for neuroanatomy and neurogenetics in drosophila. *Proc. Natl. Acad. Sci.* **105**, 9715–9720 (2008).
8. J Schindelin, et al., Fiji: An open-source platform for biological-image analysis. *Nat. Methods* **9**, 676–682 (2012).
9. A Iyengar, J Imoehl, A Ueda, J Nirschl, CF Wu, Automated quantification of locomotion, social interaction, and mate preference in drosophila mutants. *J. Neurogenetics* **26**, 306–316 (2012).
10. A Iyengar, CF Wu, Flight and seizure motor patterns in drosophila mutants: Simultaneous acoustic and electrophysiological recordings of wing beats and flight muscle activity. *J. Neurogenetics* **28**, 316–328 (2014).
11. GR Holt, WR Softky, C Koch, RJ Douglas, Comparison of discharge variability in vitro and in vivo in cat visual cortex neurons. *J. Neurophysiol.* **75**, 1806–1814 (1996).
12. J Lee, A Iyengar, CF Wu, Distinctions among electroconvulsion- and proconvulsant-induced seizure discharges and native motor patterns during flight and grooming: quantitative spike pattern analysis in drosophila flight muscles. *J. Neurogenetics* **33**, 125–142 (2019).
13. IC Howlett, MA Tanouye, Seizure-sensitivity in drosophila is ameliorated by dorsal vessel injection of the antiepileptic drug valproate. *J. Neurogenetics* **27**, 143–150 (2013).
14. JW Wang, AM Wong, J Flores, LB Vosshall, R Axel, Two-photon calcium imaging reveals an odor-evoked map of activity in the fly brain. *Cell* **112**, 271–282 (2003).
15. JE Engel, CF Wu, Interactions of membrane excitability mutations affecting potassium and sodium currents in the flight and giant fiber escape systems of drosophila. *J. Comp. Physiol. A* **171**, 93–104 (1992).
16. J Lee, CF Wu, Electroconvulsive seizure behavior in drosophila: analysis of the physiological repertoire underlying a stereotyped action pattern in bang-sensitive mutants. *The J. Neurosci.* **22**, 11065–11079 (2002).
17. K Ito, et al., A systematic nomenclature for the insect brain. *Neuron* **81**, 755–765 (2014).
